# Pyrroloquinoline quinone inhibits PCSK9-NLRP3 mediated pyroptosis of Leydig cells in obese mice

**DOI:** 10.1038/s41419-023-06162-8

**Published:** 2023-11-07

**Authors:** Jinyuan Wang, Shun Zhang, Linlin Hu, Yan Wang, Ke Liu, Jianghua Le, Yongpeng Tan, Tianlong Li, Haoxuan Xue, Yanhong Wei, Ou Zhong, Junhui He, Dan Zi, Xin Lei, Renhe Deng, Yafei Luo, Masong Tang, Mingxuan Su, Yichang Cao, Qingyou Liu, Zhihan Tang, Xiaocan Lei

**Affiliations:** 1 Clinical Anatomy and Reproductive Medicine Application Institute, Department of Histology and Embryology, Postdoctoral Station for Basic Medicine, Hengyang Medical School, University of South China, Hengyang, 421001 China; 2 Department of Reproductive Medical Center, The Affiliated Hospital of Guilin Medical University, Guilin, 541001 China; 3 Reproductive Medicine Center, The Affiliated Hospital of Youjiang Medical University for Nationalities, Baise, 533000 China; 4 State Key Laboratory for Conservation and Utilization of Subtropical Agro-Bioresources, Guangxi University, Nanning, 530004 China

**Keywords:** Mechanisms of disease, Infertility, Transcriptomics, Drug regulation, Apoptosis

## Abstract

Abnormal lipid metabolism and chronic low-grade inflammation are the main traits of obesity. Especially, the molecular mechanism of concomitant deficiency in steroidogenesis-associated enzymes related to testosterone (T) synthesis of obesity dominated a decline in male fertility is still poorly understood. Here, we found that in vivo, supplementation of pyrroloquinoline quinone (PQQ) efficaciously ameliorated the abnormal lipid metabolism and testicular spermatogenic function from high-fat-diet (HFD)-induced obese mice. Moreover, the transcriptome analysis of the liver and testicular showed that PQQ supplementation not only inhibited the high expression of proprotein convertase subtilisin/Kexin type 9 (PCSK9) but also weakened the NOD-like receptor family pyrin domain containing 3 (NLRP3)-mediated pyroptosis, which both played a negative role in T synthesis of Leydig Cells (LCs). Eventually, the function and the pyroptosis of LCs cultured with palmitic acid in vitro were simultaneously benefited by suppressing the expression of NLRP3 or PCSK9 respectively, as well the parallel effects of PQQ were affirmed. Collectively, our data revealed that PQQ supplementation is a feasible approach to protect T synthesis from PCSK9-NLRP3 crosstalk-induced LCs’ pyroptosis in obese men.

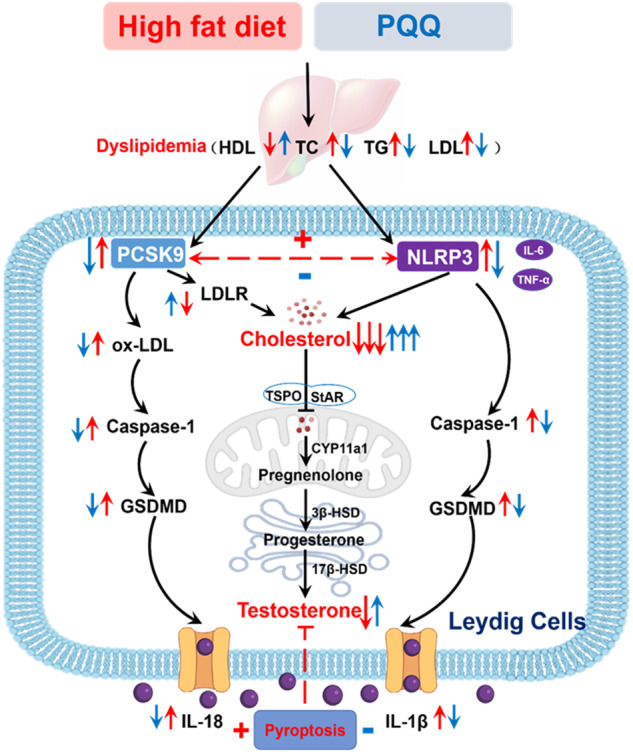

## Introduction

Obesity is a chronic metabolic disease characterized by an abnormal accumulation of body fat as well as chronic low-grade inflammation, which has been recognized as a global epidemic by WHO in the 21st century [1]. Numerous cohort studies have reported that a male factor induced by obesity contributes to infertility in approximately 25% of couples who fail to conceive [2, 3]. Obesity results in a negative impact on male reproduction potential, including erectile dysfunction, hypogonadism, and poor semen quality [4]. High-fat-diet (HFD)-induced pathologic changes in Leydig Cells (LCs) with lipid droplets and inflammatory response is the crucial mechanism for lowing testosterone (T) to the disruption of male reproductive function [5, 6]. Although studies have confirmed that alteration in the hypothalamic-pituitary-gonadal axis, insulin resistance (IR) as well as excessive accumulation of aromatase and estrogen is closed related to obesity-induced T deficiency [7, 8], recent progress reports that proprotein convertase subtilisin/kexin type 9 (PCSK9) inhibits the uptake of cholesterol for T synthesis, meanwhile, its upregulation was significantly negatively correlated with sex hormone-binding globulin (SHBG) secretion in obesity [9]. While PCSK9 became one of the most promising targets for the improvement of hypercholesterolemia, as for its inhibitors constituted a new class of lipid-lowering drugs [10, 11], whether PCSK9 participates in regulating T synthesis remains unknown.

Nowadays, evidence is apparent that obesity-induced activation of inflammatory cytokines (e.g., NLRP3, IL-1β, IL-18) is a major culprit behind the adiposity-related metabolic complication [12, 13]. Especially, the accumulation of NLRP3 promoted obesity-related impairment of spermatogenesis and testosterone synthesis by triggering interleukin-1β (IL-1β) secretion [14]. Furthermore, pyroptosis in LCs mediated by NLRP3/Caspase-1/GSDMD (Gasdermin D) pathway is also essential to the pathogenesis of sterile inflammatory diseases such as Cd exposure [15, 16], whether obese-associated pyroptosis of LCs leads to the dysfunction of T synthesis needs to clarify. Simultaneously, PCSK9 activates NLRP3 inflammasome signaling (NLRP3, Caspase-1, IL-1β and IL-18), subsequently induces Caspase-1-dependent pyroptosis in chronic myocardial ischemia [17], which in turn NLRP3 inflammasome could activate PCSK9 secretion in cardiac endothelial cells of obese mice [18]. Therefore, it is of great significance to explore the relationship between the PCSK9-NLRP3 crosstalk and the pathogenic mechanism of testosterone synthesis disorder in obesity.

Pyrroloquinoline quinone (PQQ) is a newly discovered oxidoreductase coenzyme, exerting potential health benefits in anti-diabetic, anti-oxidative, and neuroprotective actions and so on [19]. Study has demonstrated that PQQ could alleviate the hyperlipidemia-generated lipid accumulation with the reduced levels of TC and TG in liver or plasma [20]. Besides, PQQ also could protect hepatocyte from lipotoxicity and inflammation followed obesity via down-regulating the level of pro-inflammatory cytokines (NLRP3, IL-6, TNF and IL-1β) [21]. Additively, PQQ possesses pharmacological effects on mitigating NLRP3 inflammasome-mediated pyroptosis to block the progression of inflammatory disease such as diabetes and obesity [22–24]. In the present study, we discovered that PQQ restored the lipid metabolism, T synthesis and semen quality in HFD-induced obese mouse models in vivo, which was characterized the essential functions of PCSK9 and NLRP3. Furthermore, we ascertained that PQQ improved the T synthesis by increasing cholesterol intake and suppressing the pyroptosis of LCs in vitro, which functioned by inhibiting the expression of PCSK9 and NLRP3 respectively. Together, our study defines a previously unknown function and molecular mechanisms in which PQQ intervention effectively ameliorates male reproductive dysfunction.

## Results

### PQQ ameliorates the abnormal lipid metabolism in obese mice

**Fig. 1:**
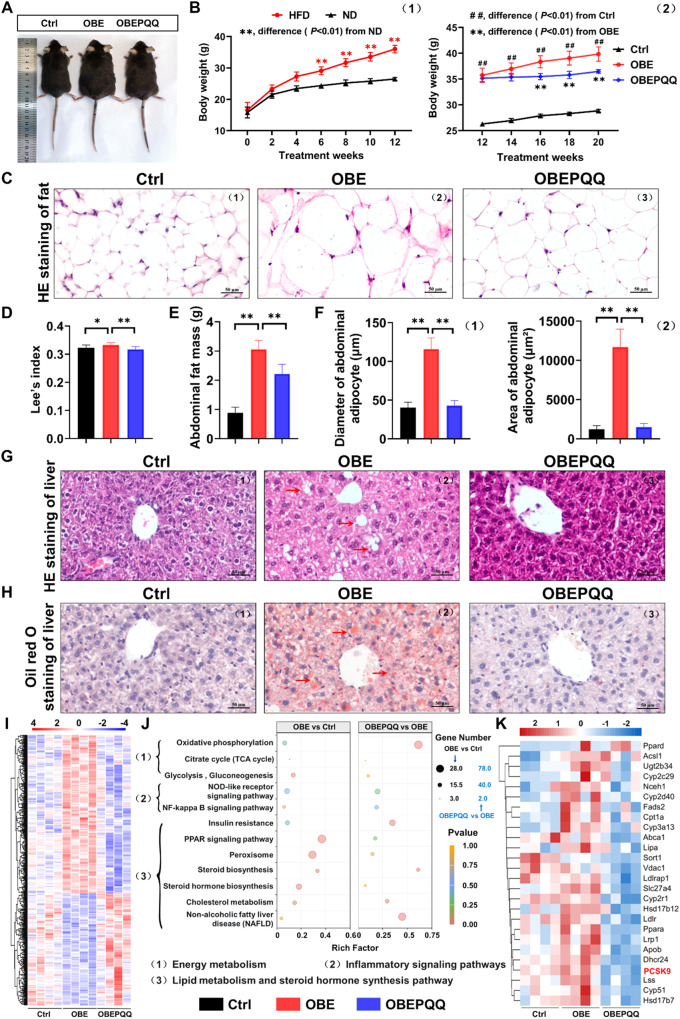
PQQ ameliorates the abnormal lipid metabolism in obese mice. **A** Representative appearance of mice at end of the study. **B** Comparison between the body weight of mice in the HFD and ND groups (1), and change in body weight in response to each diet and treatment (2) (*n* = 12). **C** The representative light microscopic histologyimages of abdominal adipose masses stained with hematoxylin and eosin (HE). **D** Relative LEE’s index. **E** Abdominal fat mess weight (*n* = 12). **F** The diameter (1) and area (2) of abdominal adipocyte in the mice. **G** HE staining of liver. **H** Hepatic lipid droplets were visualized by oil red O staining. The red arrow indicates fat droplet. **I** Cluster analysis of differentially expressed genes (DEGs) which are consistently effective treatment of PQQ enriched in mice’s liver (*n* = 4). **J** KEGG Functional enrichment analysis of DEGs. **K** Cluster analysis of DEGs enriched in lipid metabolism and steroid hormone synthesis pathway. *P < 0.05, **P < 0.01.

To preliminary address whether PQQ supplementation would restore obesity, the body and abdominal fat weight as well as the levels of lipid metabolism of liver in three group were investigated to evaluate the effectiveness of PQQ. As expected, a significant increase in weight gain of average body, liver and abdominal fat was detected in obese mice compared with normal ones (Fig. 1A, B, E, Fig. S1C). In contrast, besides improvement on body, liver and abdominal fat weight, administration of PQQ in obese mice also remarkably attenuated the Lee’s index (0.324 ± 0.011, 0.333 ± 0.008, 0.317 ± 0.009, respectively) and enlarged diameter (1225.99 ± 448.29 µm^2^, 11707.39 ± 2235.57 µm^2^, 1505.99 ± 448.20 µm^2^, respectively) and area (40.27 ± 6.97 µm, 115.77 ± 14.39 µm, 42.89 ± 6.56 µm, respectively) of abdominal adipocytes gained in HFD-fed (Fig. 1C, D, F). Besides, PQQ is a potential therapeutic agent for the treatment of the HFD-impaired food intake and glucose tolerance, as assessed by the oral glucose tolerance test (OGTT) (Fig. S1A, B). Since the production and degradation of fatty acid mainly takes place in peripheral organs such as the adipose tissue and liver. To investigate whether a HFD have a negative effect on the structural as well as function of the liver and whether those could be ameliorated by PQQ supplement, liver tissues were examined by HE stains (Fig. 1G) and Oil red O staining (Fig. 1H, Fig. S1D). Obviously, the size and number of lipid droplets in the liver accumulated in the HFD group, whereas PQQ administration prevented excessive lipid significantly (The quantification of NAFLD activity scores is also provided in Table S2). Transcriptome analysis was conducted to uncover the metabolic features of the mice liver following abnormal lipid metabolism. The results showed that there were 1023 upregulated and 824 downregulated genes between the OBE group and the Ctrl group, 2539 upregulated genes and 2564 downregulated genes in OBEPQQ mice compared with OBE mice (Fig. S1F), among which there were 628 differentially expressed genes (DEGs) distinctly expressed between the three groups (Fig. S1E). DEḍGs were subjected to hierarchical clustering, further highlighting clusters of genes that are adverse expression trends of obese mice with OBEPQQ and Ctrl ones (clusters 3–4, 7–8, 11, 13 and 15), which was shown in the heatmap. (Fig. S1G, Fig. 1I). Moreover, the main enriched GO terms (Fig. S1H, I) and KEGG pathways (Fig. 1J) are mainly overlapped in energy metabolism, inflammatory signaling pathways, lipid metabolism and steroid synthesis pathway. Most notably, 25 DEGs in above pathways were restored markedly in PQQ administration. The clustering tree of these DEGs showed that the expression of PCSK9, which are related to cholesterol metabolism pathway, mediating the degradation of LDL to cholesterol by lysosomes [25], were downregulated in response to OBEPQQ group (Fig. 1K). These results were consistent with the histopathological changes in liver tissue caused by HFD. All of results above indicate that abnormal lipid metabolism occurred in male mice fed with chronic HFD, and that could be alleviated by PQQ intervention.

### PQQ improves the abnormalities in serum lipid metabolism in obese mice

**Fig. 2:**
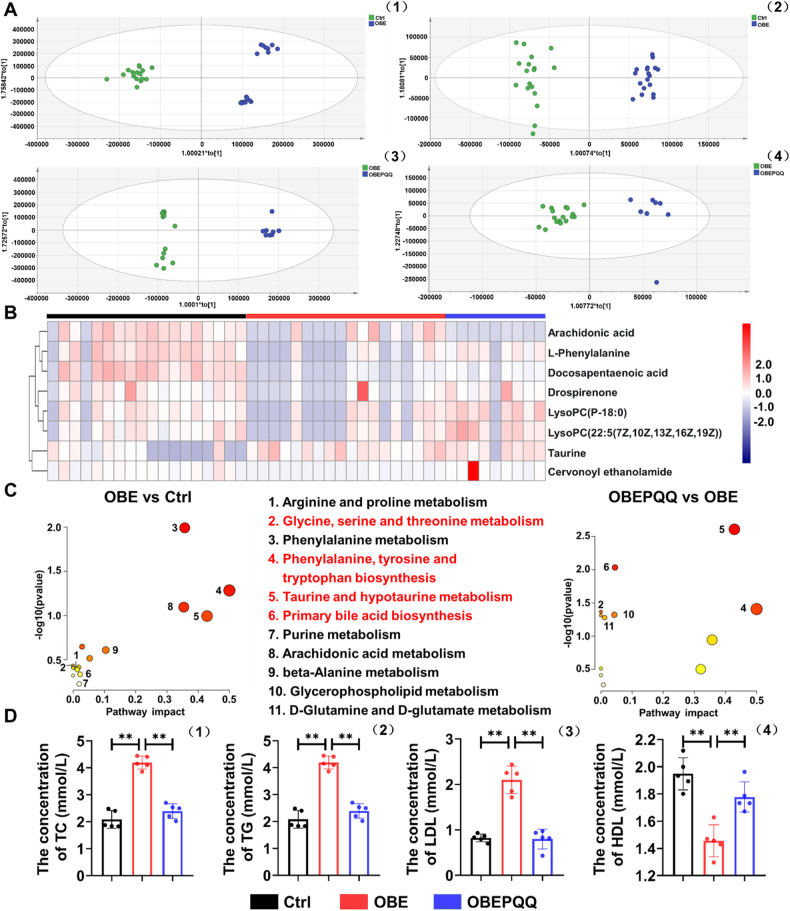
PQQ improves the abnormalities in serum lipid metabolism in obese mice. **A** Orthogonal partial least square discriminant analysis (OPLS-DA) score plots of Ctrl and OBE group showed clear distinction in the positive ion mode (1) and the negative ion mode (2). OPLS-DA score plots of OBE and OBEPQQ group showed clear distinction in the positive ion mode (3) and the negative ion mode (4) (Ctrl, *n* = 18; OBE, *n* = 18; OBEPQQ, *n* = 8). **B** Heatmap of the changed metabolites about lipid metabolism identified in serum samples analyzed. **C** Summary plot for computed metabolic pathway analysis of differential metabolites identified pathways as a function of log(p) (y-axis) and the pathway impacts of the key metabolites (x-axis) that differed between OBE and Ctrl group, OBEPQQ and OBE group. **D** Analysis of serum TC level (1), TG level (2), LDL level (3), and HDL level (4). *P < 0.05, **P < 0.01 (*n* = 5).

To identify distinct metabolites that may be associated with abnormal lipid metabolism in obesity among thousands of variables, a pairwise comparison was conducted between the various group via analysis of serum metabolomics. PCA score and OPLS-DA supervised model indicate stable and reliable differentiation between the groups, with the detailed parameters showed in Supplementary Table 1 (Fig. 2A, Fig. S2A, B). The generated cluster heatmap of the changed metabolites identified in three groups showed that similar metabolites were located in close proximity, indicating that the samples of the Ctrl and OBE groups can be separated, with the pattern of OBEPQQ group consistent with Ctrl group (Fig. 2B). As for the metabolic pathway analysis, a total of 16 pathway were observed for the main metabolic pathway between Ctrl and OBE group among which 1 pathway with *P* < 0.05; 12 between OBE and OBEPQQ group, among which 6 pathway with *P* < 0.05 (detailed information can be found in Supplementary Tables 3 and 4), including pathways was identified in all three groups: Glycine, serine and threonine metabolism, Phenylalanine, tyrosine and tryptophan biosynthesis, Taurine and hypotaurine metabolism, Primary bile acid biosynthesis (Fig. 2C), and the last three pathways association with cholesterol degradation. Furthermore, the level of serum lipid metabolizing hormones has been analyzed. As shown in Fig. 2D, the TC, TG and LDL contents in the HFD group increased compared with those in the control group and all of those reduced significantly by PQQ supplement. On the contrary, the lower levels of HDL were observed in serum of HFD-induced obese mice. While this tendency reversed following PQQ treatment. The results demonstrated that PQQ administration may played vital roles in the improvement effect of attenuating abnormal cholesterol metabolism on obese mice. The underlying mechanism may be dependent on the primary bile acid biosynthesis, and then improve the lipid metabolism abnormalities in serum.

### PQQ attenuates the testicular dysfunction in obese mice

**Fig. 3:**
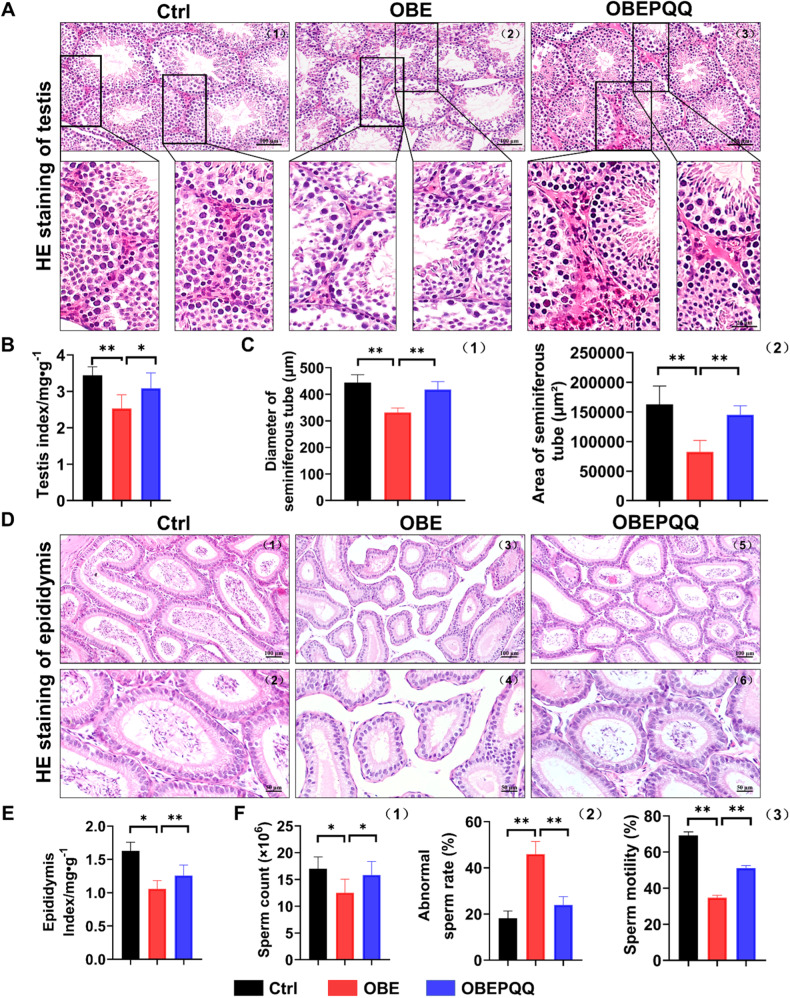
PQQ attenuates the testicular dysfunction in obese mice. **A** HE staining of testis. **B** The testis index of mice (*n* = 10). **C** The diameter (1) and area (2) of seminiferous tube in the mice. **D** HE staining of epididymis. **E** The epididymis index of mice (*n* = 10). **F** The sperm count (1) (*n* = 10), abnormal sperm rate (2) (*n* = 11) and sperm motility (3) (*n* = 6) at the end of the experiment. *P < 0.05, **P < 0.01.

To establish the harmful effects of HFD on male reproductive function, we analyzed their pathological change on the testes of HFD-induced obese mice. Morphological analysis found that PQQ effectively protected testes structures against HFD damage and reduced the loss of spermatogenic cells and LCs (Fig. 3A), increasing the diameter (Ctrl: 444.64 ± 28.47 µm; OBE: 332.07 ± 16.50 µm and OBEPQQ: 417.842 ± 29.56 µm respectively, being significantly different and *P* < 0.05) and area(Ctrl: 162794.28 ± 30088.52 µm2; OBE: 82663.17 ± 18558.00 µm2 and OBEPQQ: 144810.51 ± 15152.32 µm2 respectively, being significantly different and *P* < 0.05) of seminiferous tubule, which was reduced by feeding HFD (Fig. 3C). The epididymal ducts of Ctrl and OBEPQQ mice which was rich in sperm are well-formed, tightly spaced. Whereas a few sperm can be observed in the abnormally shaped epididymis of OBE mice (Fig. 3D). Meanwhile, in contrast to the Ctrl group, PQQ supplement significantly improved the ratio of testes/body weight and epididymis/body weight in obese mice (Fig. 3B, E). Next, the effects of PQQ on sperm characteristics was assessed. The sperm concentration which was regard as an indicator of fertilization potential was found to be obviously decreased in the OBE group mice, while PQQ treatment reversed this effect (Fig. 3F). Likewise, PQQ was shown to reduce the number and percentage of abnormal sperm induced by the HFD (Fig. 3F). Furthermore, Compared with Ctrl group, mice in the OBE group showed a remarkable decrease in motile sperm and the improvement in response to PQQ treatment significantly (Fig. 3F). Taken together, those data showed that the protective effect of PQQ on testicular injury.

### PQQ improves the impaired testosterone synthesis in obese mice

**Fig. 4:**
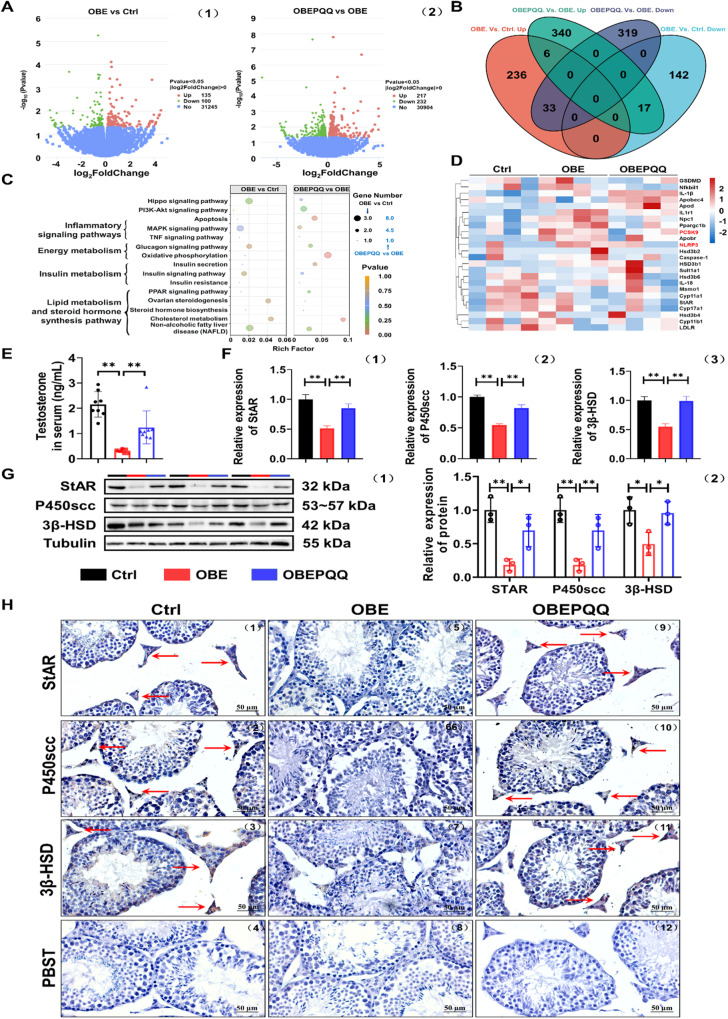
PQQ improves the impaired testosterone synthesis in obese mice. **A** Volcano plots of testicular DEGs in OBE vs. Ctrl groups (1) and OBEPQQ vs. OBE groups (2) (*n* = 4). **B** The venn diagram of DEGs. **C** The functional enrichment analysis of DEGs. **D** The heatmap of the changed DEGs identified in functional enrichment analysis. **E** The level of testosterone in serum (*n* = 8). **F** qRT-PCR validation of StAR, P450scc and 3β-HSD in testes (*n* = 3). **G** Expression of StAR, P450scc and 3β-HSD in testes detected by WB analysis (1) and quantified by ImageJ software (2). Relative quantitation of protein level normalized to tubulin (*n* = 3). **H** Immunohistochemistry analysis of the expression of StAR, P450scc and 3β-HSD in testes. PBST was used as the negative control. The red arrows indicate the cells with positive signal. *P < 0.05, **P < 0.01.

Subsequently, testicular transcriptomics was exerted to explore the potential toxic mechanism of HFD-induced male reproductive injury and reverse effect of PQQ. Compared with the controls, there were 275 upregulated and 159 downregulated DEGs in OBE mice; there were 363 upregulated and 352 downregulated DEGs between the OBEPQQ group and the OBE group (Fig. 4A). Altogether, 56 genes were differentially expressed between the three groups, among which 50 DEGs are adverse expression trends of obese mice with OBEPQQ and Ctrl ones (Fig. 4B). We further performed a function enrichment analysis based on the KEGG and GO database (Fig. 4C, Fig. S3B, F). Most notably, DEGs were significantly enriched in the metabolic pathways such as inflammatory signaling pathways, energy metabolism and insulin metabolism pathway. In addition, 24 DEPs in above pathways were restored observably in PQQ administration. Similar to the analysis of liver transcriptomics, the clustering tree of these DEGs showed that proteins associated with cholesterol metabolism such as PCSK9, and pyroptosis-related proteins (e.g., NLRP3, Caspase-1, and GSDMD) were upregulated, LDLR and steroid synthesis-associated proteins P450scc and 3β-HSD were downregulated in response to HFD, which could be alleviated by PQQ intervention (Fig. 4D).

Since our experimental results have confirmed that HFD damage male reproduction to obese mice mainly stemming from the inhibition of steroid metabolism. Further studies focused on the level of T and the key enzyme for T synthesis which was often coupled with T deficiency. As is exhibited in Fig. 4E, PQQ intervention significantly enlarged the T content in serum from obese mice. The expression level of StAR, P450scc and 3β-HSD, an important T synthesis-associated gene/protein (enriched in lipid metabolism and steroid hormone synthesis pathway in both GO and KEGG analyses), were then determined by qRT-PCR, WB and IHC analysis (Fig. 4F–H, Fig. S3C), showing the same pattern with testicular transcriptome results. Our results suggested that PQQ improves the level of T may thanks to relieving the impaired expression of the key enzyme gene/protein for T synthesis in obese mice.

### PQQ inhibits the pyroptosis of Leydig cells in obese mice

**Fig. 5:**
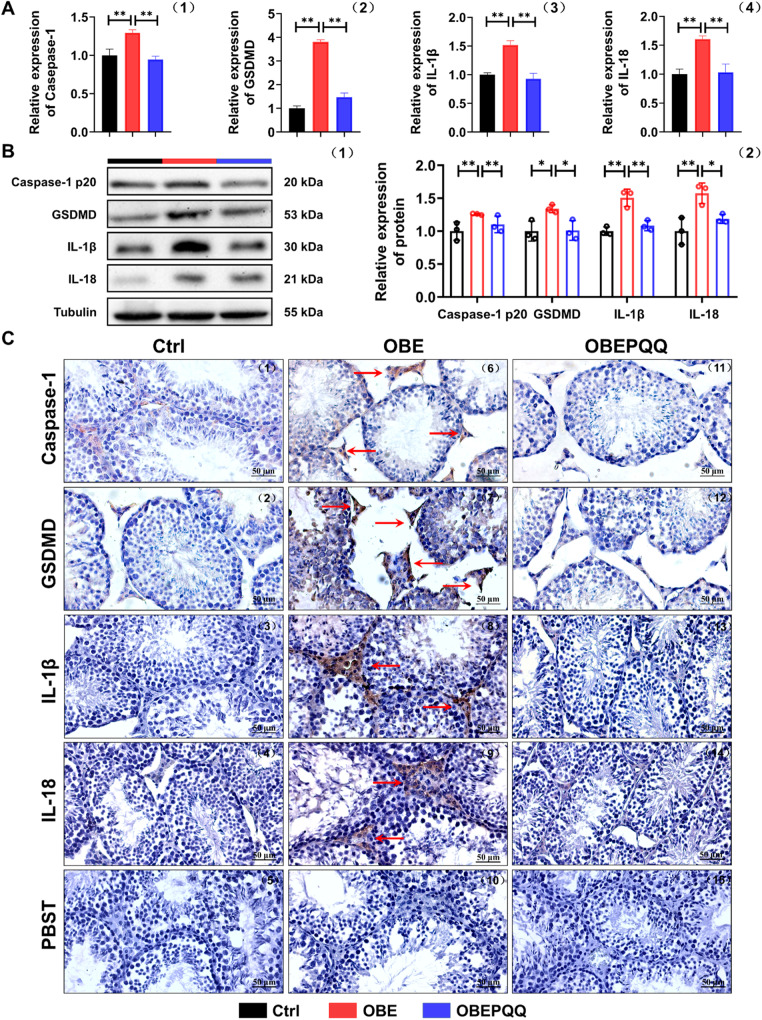
PQQ inhibits the pyroptosis of Leydig cells in obese mice. **A** qRT-PCR validation of Caspase-1, GSDMD, IL-1β and IL-18 in testes (*n* = 3). **B** Expression of Caspase-1 p20, GSDMD, IL-1β and IL-18 in testes detected by WB analysis (1) and relative quantified by ImageJ software (2). Relative quantitation of protein level normalized to tubulin (*n* = 3). **C** Immunohistochemistry analysis of the expression of Caspase-1, GSDMD, IL-1β and IL-18 in testes. PBST was used as the negative control. The red arrows indicate the cells with positive signal. *P < 0.05, **P < 0.01.

Compelling evidence addressed that pyroptosis were reported to subsequently trigger toxic substances-related injury in teste [26]. An assumption we came up with that a HFD could induce the pyroptosis of LCs, leading to damaged T synthesis, and it is where the therapeutic effects of PQQ interventions to improve male reproduction. Hence, the expression level of pyroptosis factors (Caspase-1 p20, GSDMD, IL-1β and IL-18) which were enriched in the inflammatory response in both GO and KEGG analyses, were then determined by qRT-PCR, WB and IHC analysis (Fig. 5F–H, Fig. S3D). Coinciding with Fig. 4D, a sharply increased of above factors was observed in LCs of obese mice compared with the Ctrl group, nevertheless, the expressions of which could be significantly down-regulate after PQQ treatment. Altogether, our results confirm that PQQ ameliorates low T in obese mice via inhibiting the HFD-induced pyroptosis of LCs.

### PQQ protects against testicular injury by regulating PCSK9 and NLRP3

**Fig. 6:**
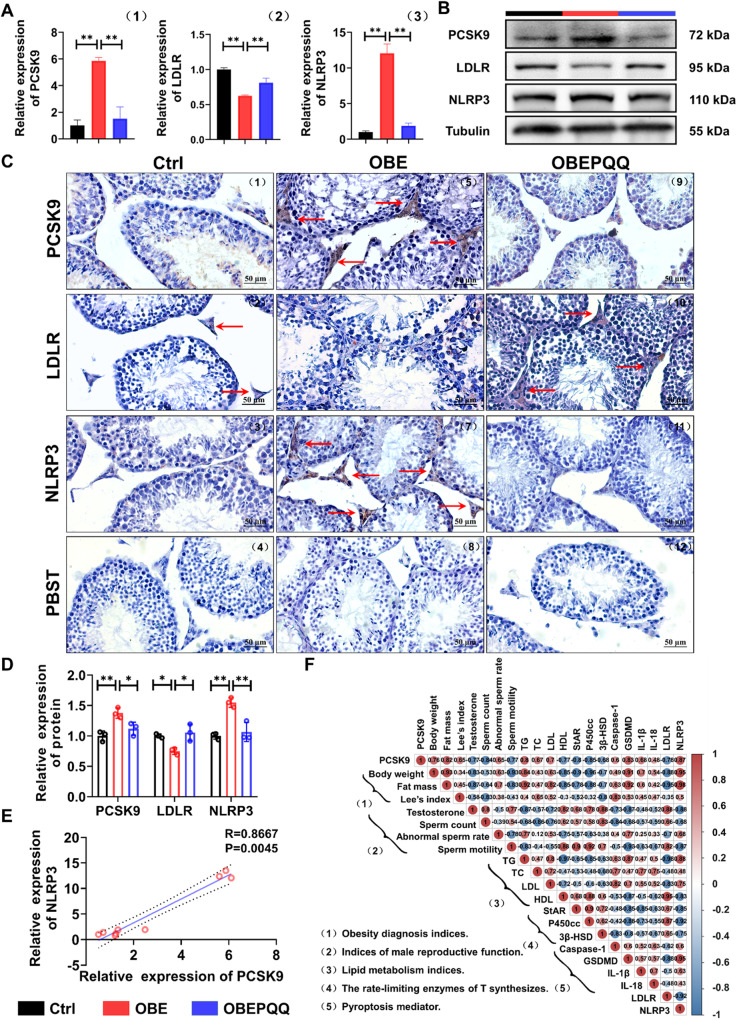
PQQ protects against testicular injury by regulating PCSK9 and NLRP3. **A** The mRNA levels of PCSK9, LDLR and NLRP3 in testes analyzed by qRT-PCR (*n* = 3). **B** The protein levels of PCSK9, LDLR and NLRP3 in testes were determined by WB analysis (*n* = 3). **C** Immunohistochemistry analysis of the expression of PCSK9, LDLR and NLRP3 in testes. PBST was used as the negative control. The red arrows indicate the cells with positive signal. **D** The quantified analyses of WB (Fig. 7B) by ImageJ software. Relative quantitation of protein level normalized to tubulin. **E** Spearman’s correlation coefficient analysis between the PCSK9 and NLRP3. **F** Correlation coefficient matrix showing the functional correlation between the altered expression of PCSK9-NLRP3 pathway and the indicators of testosterone synthesis disorders; Correlation analysis was performed using Spearman’s correlation due to a skewed distribution of the data. The value of r represents the degree of correlation (0 > r > 1, positive correlation; −1 < r < 0, negative correlation). *P < 0.05, **P < 0.01.

To further clarify the correlations which lead to insufficient T synthesis in obesity between the disorders of cholesterol metabolism and pyroptosis of LCs, elucidating the mechanism of PQQ improving male reproductive dysfunction, the expression of PCSK9 and NLRP3 that subsequently trigger to pyroptosis [27, 28] has been determined in testes at first. LCs have the capacity to biosynthesize testosterone from cholesterol in testicular spermatogenic function [25], whereas dysregulation of PCSK9 which targeted LDLR for subsequent degradation leading to hypercholesterolemia and decreasing the level of intracellular cholesterol [29]. As expected, agree with the transcriptome results of testes, an increase of PCSK9 and NLRP3 specifically expressed in LCs of obese mice detected by qRT-PCR, WB and IHC (Fig. 6A–D, Fig. S3E), and recovery of values in response to PQQ treatment. Interestingly, we next examined that the expression of LDLR, a downstream signaling pathway target of PCSK9, is actually reduced in mice fed HFD which could be suppressed via PQQ administration (Fig. 6A–D).

In addition, it was reported that there was a positive correlation between the level of NLRP3 inflammasome and PCSK9 secretion particularly in the presence of HFD [18]. To further verify our hypothesis, finding the key communities of PCSK9-NLRP3 interaction associated with male reproductive dysfunction induced by obesity, Spearman’s correlation analysis was carried out to analyze the links between PCSK9 and NLRP3, indicating a positively significant correlation (Fig. 6E). Next, we therefore identified the correlation of indices of obesity diagnosis, male reproductive function, lipid metabolism indices, the rate-limiting enzymes of T synthesizes, pyroptosis mediator, respectively. the PCSK9 and NLRP3 level which was upregulated showed a significant negative correlation with the levels of male reproductive function (T, sperm count and sperm motility), T-converting proteins (StAR, 3β-HSD, P450scc), HDL and LDLR. On the contrary, the relative abundance of PCSK9 and NLRP3 positively correlated with obesity diagnosis indices (body weight, fat mass and Lee’s index), abnormal sperm rate, pyroptosis markers including Caspase-1, GSDMD, IL-1, IL-18 and the level of lipid metabolizing hormone (TG, TC and LDL) (Fig. 6F).

Thus, PCSK9-NLRP3 interrelation caused the alterations of lipid metabolism in liver as well as LCs-regulated T synthesis, which probably caused by aggravated testicular pyroptosis and insufficient cholesterol uptake in LCs of obese mice. We concluded from these observations that PQQ may be an inhibitor of LCs’ pyroptosis which was influenced by PCSK9-NLRP3 crosstalk, improving the T synthesis of obese male. However, detailed experimental evidence should be provided to support this hypothesis.

### PQQ promotes T synthesis via suppressing PCSK9-induced pyroptosis in LCs

**Fig. 7:**
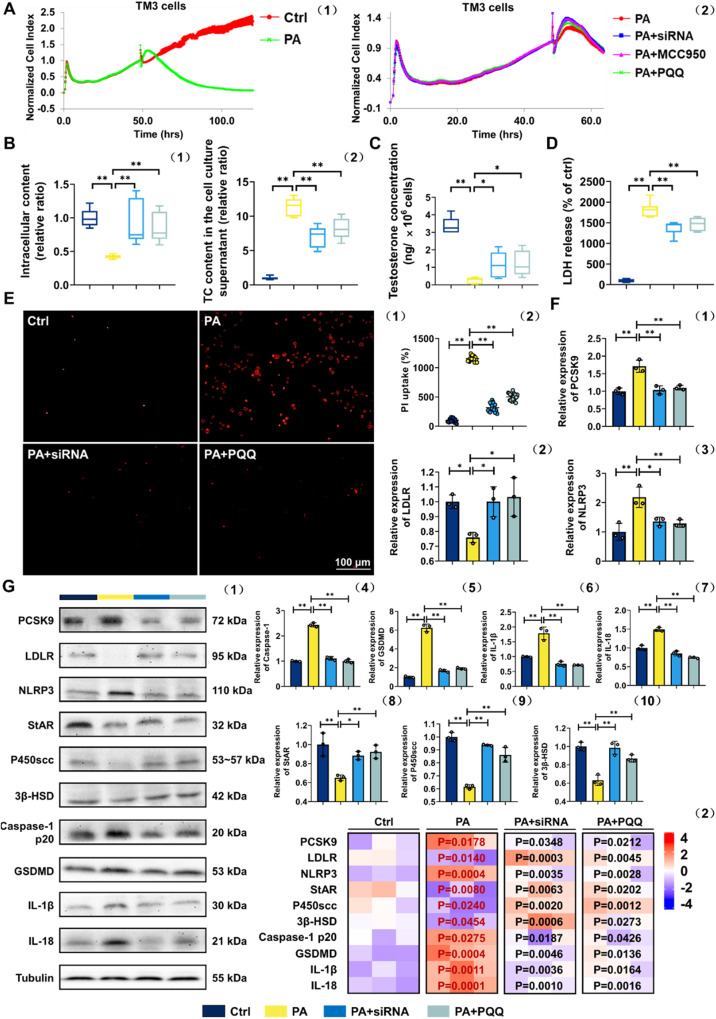
PQQ promotes T synthesis via suppressing PCSK9-induced pyroptosis in LCs. **A** Normalized cell index of TM3 cells incubated with or without 0.4 mM PA (1) and 0.4 mM PA-treated TM3 cells incubated with or without 50 nM PCSK9 siRNA, 100 nM MCC950 or 100 nM PQQ (2). Data were acquired by RTCA (*n* = 3). **B** Cholesterol levels in intracellular (1) (*n* = 9) and TM3 cells culture supernatant (2) (*n* = 8). **C** Testosterone levels of TM3 cells (*n* = 8). **D** LDH release was assayed by the LDH Cytotoxicity Assay Kit (*n* = 10). **E** The fluorescence intensity of PI was detected by a microplate absorbance reader (1) and the cells with positive signal was assessed by statistical analysis (2). **F** qRT-PCR validation of PCSK9, LDLR, NLRP3, StAR, P450scc, 3β-HSD, Caspase-1, GSDMD, IL-1β and IL-18 (*n* = 3). **G** Expression of PCSK9, LDLR, NLRP3, StAR, P450scc, 3β-HSD, Caspase-1 p20, GSDMD, IL-1β and IL-18 detected by WB analysis (1), and heatmap showing relative quantified analysis performed by ImageJ software (2). Relative quantitation of protein level normalized to tubulin; The red one indicates *p*-value vs Ctrl group, while the black one indicates *p*-value vs PA group; ns means no significant with Ctrl group. *P < 0.05, **P < 0.01. (*n* = 3).

To corroborate whether PQQ amelioration of T synthesis dysfunction in obese mice derived from the suppression of PCSK9, which gives rise to NLRP3-maneged pyroptosis of LCs, we used siRNA duplexes or selective inhibitor MCC950 [30] to knock down the expression of PCSK9 or NLRP3 respectively in a PA-treated TM3 cell model [31]. Based on the detection of cell viability analysis and expression of genes, 0.4 mM PA, 100 nM MCC950 as well as 100 nM PQQ was selected as optimal drug-given concentrations for the experiments that followed, with 50 nM PCSK9 siRNA successfully silencing the PCSK9 gene (Fig. S4A, F, G, *P* < 0.05). PA treatment exhibited cytolysis of TM3 cells, which could be significantly improved by PCSK9 siRNA, MCC950 and PQQ intervention according to RTCA monitoring, indicating that the effectiveness of PQQ administration specifically target PA (Fig. 7A).

We also directly detected the presence of the T and cholesterol both in intracellular and cell culture supernatant. Briefly, T was highly abundant in PA-treated TM3 cells after PQQ management, with higher intracellular cholesterol as well as lower extracellular cholesterol (Fig. 7B, C). As anticipated, Fig. 7D shows that the LDH release induced by PA was significantly reduced after PCSK9 inhibition and PQQ administration. The uptake of PI was used to quantitatively examine membrane damage in individual cells during diverse interventions exposure, revealing that incubation of TM3 cells with PA resulted in an increase in PI uptake but recovered following PQQ supplementation (Fig. 7E, Fig. S5B). Likewise, all the effective efforts with PQQ intake above was in line with those of PA+siRNA group.

Next, similar to the efficiency of silencing PCSK9, PQQ administration were almost abolished the adverse effect induced by PA treatment, reflected in the up-expression of the LDLR and T-converting proteins (StAR, 3β-HSD, P450scc) with the downregulated expression of pro-pyroptosis factors (NLRP3, Caspase-1 p20, GSDMD, IL-1β and IL-18) (Fig. 7F, G). Notably, inhibition of PCSK9 in normal TM3 cells also plays a role in increased T synthesis by suppressing the pyroptosis partly (Fig. S4A–F, Fig. S5A). Thus, PQQ could ameliorate T synthesis in obese mice via the regulation of PCSK9, through increased cholesterol intake caused by LDLR upregulated, as well as diminished NLRP3-maneged pyroptosis of LCs.

### PQQ promotes T synthesis via suppressing NLRP3-induced pyroptosis in LCs

**Fig. 8:**
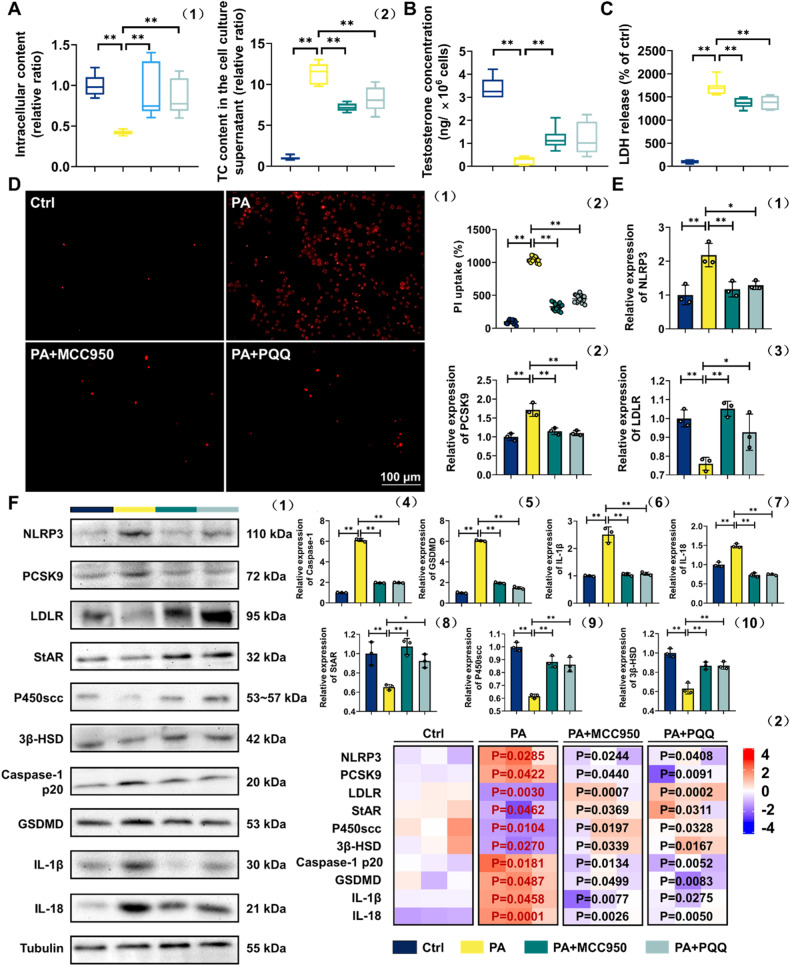
PQQ promotes T synthesis via suppressing NLRP3-induced pyroptosis in LCs. **A** Cholesterol levels in intracellular (1) (*n* = 9) and TM3 cells culture supernatant (2) (*n* = 8). **B** Testosterone levels in TM3 cells (*n* = 8). **C** LDH release was assayed by the LDH Cytotoxicity Assay Kit (*n* = 10). **D** The fluorescence intensity of PI was detected by a microplate absorbance reader (1) and the cells with positive signal was assessed by statistical analysis (2). **E** qRT-PCR validation of NLRP3, PCSK9, LDLR, StAR, P450scc, 3β-HSD, Caspase-1, GSDMD, IL-1β and IL-18 (*n* = 3). **F** Expression of NLRP3, PCSK9, LDLR, StAR, P450scc, 3β-HSD, Caspase-1 p20, GSDMD, IL-1β and IL-18 detected by WB analysis (1), and heatmap of that relative quantified analysis performed by ImageJ software (2). Relative quantitation of protein level normalized to tubulin; The red one indicates *p*-value vs Ctrl group, while the black one indicates *p*-value vs PA group. *P < 0.05, **P < 0.01. (*n* = 3).

To further dissect the hypothesis that PQQ could also regulate the expression of NLRP3, weakened the PCSK9 to ameliorate the PA-damaged T synthesis, same experimental method was also conducted. As is exhibited, PA-treated TM3 cells with MCC950 or PQQ administration are flooded with T and cholesterol (Fig. 8A, B). Moreover, PQQ severely relieved LDH release and PI intake, with the decline in NLRP3 and PCSK9 as opposed to a parallel increase in the expression of LDLR (Fig. 8C–F). In addition, TM3 cells treated with PQQ exhibited a significant up-expression of the T-converting proteins (StAR, 3β-HSD, P450scc), accompanied by the downregulated expression of pro-pyroptosis factors (NLRP3, Caspase-1 p20, GSDMD, IL-1β and IL-18) (Fig. 8E, F). Likewise, all the effective efforts with PQQ intake above was in line with those of PA + MCC950 group. Those results prove that PQQ could ameliorate T synthesis in obese mice via the regulation of NLRP3, through diminished LCs pyroptosis and increased cholesterol intake caused by PCSK9 downregulation. Hence, PQQ ameliorate the obese-induced dysfunction of T synthesis caused by pyroptosis in LCs via regulating the PCSK9-NLRP3 crosstalk.

## Discussion

Obesity is a chronic systematic inflammatory disease with an abnormal accumulation of body fat, which is accompanied by a negative impact on male reproduction potential, particular including the poor levels of T [1, 4]. T produced by cholesterol uptake in LCs is the most critical steroid hormone that maintains the fertility of men [32]. However, obesity-incurred T deficiency is tightly associated with the pathological injury of hypertrophied LCs with abundant lipid accumulation, vesicles, and autophagosome-like structures containing degenerated mitochondria [5, 33, 34]. In this study, we have explored that HFD-triggered decreased T concentration with a decline in semen quality is positively correlated with the degenerated seminiferous tubules, as well as the downregulated T-converting proteins (StAR, CYP11a1, and 3β-HSD) in LCs of obese mice, which is in line with the previous studies [35–37]. Fortunately, multiple studies have confirmed that weight loss by lifestyle intervention, bariatric surgery, or drug intervention delivered a significant amelioration of T synthesis and improvement of the quality of sexual function [38, 39]. PQQ, as a newly discovered oxidoreductase coenzyme, performed forceful therapeutic promise effectively to lose weight, combating hyperglycemia, hyperlipemia, and increased insulin levels [40]. We were pleasantly surprised that PQQ administration not only significantly lost weight but also ameliorated the damage to the structure and function of the testis, as well as T level and secretion in obese mice and PA-treated LCs. Subsequently, the biological functions of PQQ were preliminarily proven by up-regulating the T-converting proteins (StAR, P450scc, and 3β-HSD), which was identical with the other antioxidants like melatonin, lycopene, catechin and so on [41–45]. Significantly, it was the first research for PQQ combating dysfunction of T synthesis, however, the underlying mechanism of which needs to be further explored.

In order to get deeper into the molecular mechanisms that how PQQ improves the synthesis of T and spermatogenesis, obesity-associated dysfunction of lipid metabolism has to be considered as the prominent inducement [34]. Abundant cross-sectional and prospective studies have attested a significantly negative correlation between the dyslipidemia (e.g., increased level of TC, TG, LDL) and decreased free T and SHBG levels in overweight men, as for the dyslipidemia has become an independent risk factor for suppressive T-converting proteins (StAR and P450scc) levels in LCs [46–48]. In many cases, losing weight through bariatric surgery and drug intervention could prevent dyslipidemia, which delivered a significant amelioration of T synthesis [38, 39]. During all the mechanisms that regulate the dyslipidemia, PCSK9 inhibition has been verified as a biological target in enhancing serum cholesterol clearance, companying with improving the hypercholesterolemia of obese patients [10, 49, 50]. Molecular elucidation of PCSK9 inhibitors promote the blocking of low-density lipoprotein receptor (LDLR) degradation and increase LDL uptake of the liver in order to regulate intracellular and plasma cholesterol levels [51–54]. The increased expression of LDLR in LCs boosts the intake of exogenous cholesterol which as the raw material of T synthesis [55], however the function of LDLR was inhibited by HFD-induced obesity, which might influence normal spermatogenesis [56–58]. Increased PCSK9 expression induced by dyslipidemia inhibited SHBG synthesis in the liver [9], which indicated that PCSK9 may be the core of obesity-related abnormal T synthesis. PQQ has significant efficacy in losing weight and improving lipid metabolism as other antioxidants [59]. Early PQQ supplementation has persistent long-term protective effects on alleviating hyperlipidemia following decreased TG, TC and LDL in obese mice and patients [21, 60, 61]. While reliable experimental data we initially applied to confirm that PQQ administration not only effectively mediated damaged lipid and cholesterol metabolism with an increased level of intracellular cholesterol, but also depressed the expression of PCSK9, up-regulating downstream target LDLR and the T-converting proteins in the LCs, therefore, which may be the potential mechanism where PQQ improves the obesity-incurred dysfunction of T synthesis.

It is well known that obesity-triggered chronic low-grade inflammation negatively affected the male reproductive system, following disruption of semen parameters and testicular steroidogenesis with decreased key steroidogenic enzymes (StAR, P450scc, 3β-HSD and 17β-HSD) under the increasing concentrations of inflammatory cytokines (IL-1, IL-6, and TNF-α) in the testis [62, 63]. However, inhibition of inflammatory responses generated by antioxidants (like melatonin, vitamin K and fucoxanthin) was confirmed to increase T levels [64–67]. Considerable researches have proved that PQQ has a positive effect on inflammatory diseases such as obesity, diabetes, and sepsis via the mediation of pro-inflammatory cytokine such as IL-6, IL-1β and TNF-α [68–70]. Conformably, supported by Fan W’s efforts and our transcriptome analyses, we have detected that increased pro-inflammatory cytokine NLRP3 accompanying the inhibited T synthesis [71], which was alleviated in PQQ-treated obese mice. Owing to the pyroptosis mediated by NLRP3/Caspase-1/GSDMD is also an essential pathway to the pathogenesis of inflammatory-related male infertility such as male genital tract diseases and abnormal spermatogenesis [72, 73], we speculate whether the improvement of PQQ promote T synthesis came into play in management of the LCs pyroptosis induced by obesity. Applying MCC950 (a direct inhibition of NLRP3), we provide in vitro evidence documented that PQQ supplementation exerts suppression of the LCs pyroptosis, recovering the T synthesis with sufficient intracellular cholesterol levels in PA-cultured TM3 cells. Collectively, these findings initially validate the functional role of PQQ in maintaining T synthesis against NLRP3-targeted LCs pyroptosis in obese mice.

Interestingly, PCSK9 is also considered as an pro-inflammatory molecule and its inhibitors may be manifested by the downregulation of inflammatory cascade [74, 75]. In the previous reports, PCSK9 and pyroptosis signaling are highly expressed in patients with myocardial infarction [17]. PCSK9 played an important role in activating the NLRP3 pyroptosis signaling (NLRP3, Caspase-1, IL-1β, and IL-18), while PCSK9 − /− significantly suppressed expression of NLRP3 inflammasome signaling, GSDMD-NT, and LDH release [17]. Coincidentally, PCSK9 overexpression induced pyroptosis and increased ROS production in Human Umbilical Vein Endothelial Cells, which could be blocked by PCSK9 interference [76]. On the contrary, NLRP3 inflammasome with the downstream signals Caspase-1, IL-1β and IL-18 all participate in PCSK9 secretion, lower expression of NLRP3 and attenuated the secretion of PCSK9 in IL-1β-deficient mice fed with HFD [18]. In addition, NLRP3 knockout diminished pyroptosis and LDL levels, increased serum HDL levels in mice fed HFD, which along with blocked PCSK9 levels and increased LDLR concentration [77, 78]. In this study, we first demonstrated a positive correlation between the expression of PCSK9 and NLRP3 in the testis of obese mice, which both activated the pyroptosis and the increased LDL levels simultaneously in the obese mice and PA-treated TM3 cells, consistent with the previous reports [18, 79]. Furthermore, in keeping with the former researchers [80, 81], the restrained PCSK9 or NLRP3 revealed a synergistic inhibition on the pyroptosis and its related factors. Moving forward, we newly identified, for the first time, a molecular mechanism that PCSK9-NLRP3 crosstalk-induced the pyroptosis of LCs and deficiency of T synthesis can be effectively rescued by PQQ. In summary, we have originally proved the new pharmacological effects of PQQ, which could ameliorate the obesity-related male subfertility via regulating PCSK9-NLRP3 crosstalk.

### Limitations of the study

Although our study provides evidence that PQQ ameliorated obesity-related dysfunction of T synthesis via regulating PCSK9-NLRP3 crosstalk, there are several aspects that remain to be determined. Although we used one dietary model and one kind of fatty acids to induce obesity and simulate the high-fat environment, validating the in vivo or in vitro activity of LCs, availability of human LCs line may provide more relevance to the human obesity-induced male infertility. Future mechanistic studies should pay more attention to whether the same pathogenic mechanism that PCSK9-NLRP3 crosstalk triggering LCs pyroptosis exists in obesity-induced male infertility results from various pathogenic factors.

## Materials/subjects and methods

### Key resources table

See Table 1 for key resources information.

**Table 1:** Key resources.

Reagent or resource	Source	Identifier
Antibodies		
Rabbit polyclonal anti-PCSK9 (IHC: 1:200, WB: 1:1000)	ABclonal	Cat# A7860, RRID:AB_2770818
Rabbit polyclonal anti-LDLR (IHC: 1:150, WB: 1:1000)	Abmart	Cat# T55235; N/A
Rabbit polyclonal anti-NLRP3 (IHC: 1:150, WB: 1:1000)	ABclonal	Cat# A5652, RRID:AB_ 2766412
Rabbit polyclonal anti-caspase-1 (WB: 1:1000)	ABclonal	Cat# A0964, RRID:AB_2757485
Rabbit polyclonal anti-total and cleaved caspase-1 antibody (IHC: 1:150)	Abmart	Cat# PY10200; N/A
Rabbit monoclonal anti-GSDMD (IHC: 1:500, WB: 1:1000)	Abcam	Cat# ab219800, RRID:AB_2888940
Rabbit polyclonal anti-IL-1β (IHC: 1:150, WB: 1:1000)	ABclonal	Cat# A16288, RRID:AB_2769945
Rabbit polyclonal anti-IL-18 (IHC: 1:150, WB: 1:1000)	Proteintech	Cat# 10663-1-AP, RRID:AB_2123636
Rabbit polyclonal anti-StAR (IHC: 1:150, WB: 1:1000)	Immunoway	Cat# YN1369; N/A
Rabbit polyclonal anti-CYP11a1 (IHC: 1:150, WB: 1:1000)	Abmart	Cat# PU986898; N/A
Rabbit polyclonal anti-3β-HSD (IHC: 1:200, WB: 1:1000)	Immunoway	Cat# YN0349; N/A
Rabbit polyclonal anti-3β-tubulin (WB: 1:5000)	ABclonal	Cat# AC015, RRID:AB_2773007
Goat anti-rabbit IgG (H + L), HRP conjugate (WB: 1:1000)	Proteintech	Cat# SA00001-2, RRID:AB_2722564
Goat Anti-Rabbit IgG(H + L), biotin conjugate (IHC: 1:200)	Proteintech	Cat# SA00004-2, RRID:AB_2890946
Chemicals, peptides, and recombinant proteins		
High-fat-diet	Beijing Keao Xieli Feed	Cat# D12492
Pyrroloquinoline quinone	Cima Science	Cat# 72909-34-3
Palmitic acid	Kunchuang Biotechnology	Cat# SYSJ-KJ
MCC950	SparikJade	Cat# SJ-MX0058A
DMEM/F-12	Gibco	Cat# C11330500BT
Certified fetal bovine serum	VivaCell Bioscience	Cat# C04001-500
Horse Serum	VivaCell Bioscience	Cat# C2510-0500
Trypsin-EDTA solution, 0.25% (without phenol red)	Solarbio	Cat# T1300
OCT compound	Tissue Tek	Cat# 4583
Oil Red O	Sigma-Aldrich	Cat# 1320-06-5
Western blotting stripping buffer	Solarbio	Cat# SW3020
Propidium iodide staining solution	BD, Biosciences	Cat# 51-66211E
Annexin V binding buffer, 10X conc	BD, Biosciences	Cat# 51-66121E
DNase/RNase-free water	Solarbio	Cat# R1600
Trizol reagent	Solarbio	Cat# 15596-018
Triton X-100	Sigma-Aldrich	Cat# V900502
Tween 20	Sigma-Aldrich	Cat# V90054
Phosphate-buffered saline	Thermo Scientific	Cat# 20012027
Critical commercial assays		
Total cholesterol assay kit	Jiancheng (Nanjing, China)	Cat# A111-1-1
Triglyceride assay kit	Jiancheng (Nanjing, China)	Cat# A110-1-1
Low-density lipoprotein cholesterol assay kit	Jiancheng (Nanjing, China)	Cat# A113-1-1
High-density lipoprotein cholesterol assay kit	Jiancheng (Nanjing, China)	Cat# A112-1-1
Testosterone assay kit	Jiancheng (Nanjing, China)	Cat# H090-1-2
BCA protein assay kit	Thermo Scientific	Cat# 23227
SDS-PAGE gel kit	Solarbio	Cat# P1200
eECL western blotting kit	ComWin Biotech	Cat# CW0049M
DAB substrate kit (20X)	Solarbio	Cat# DA1010
One-step gDNA removal and cDNA synthesis SuperMix	TransGen Biotech	Cat# AT311-03
ChamQ universal SYBR qPCR master mix	Vazyme (Nanjing, China)	Cat# Q711-02
LDH cytotoxicity assay kit	Beyotime (Hangzhou, China)	Cat# C0016
Cell counting kit-8 (CCK8)	Biosharp	Cat# BS350A
ribo*FECT* CP transfection kit	RiboBio	Cat# C10511-1
Experimental models: organisms/strains		
C57BL/6 J male mice	Laboratory Animal Center of the University of South China	N/A
Experimental models: cell lines		
TM3	Cellcook Biotech (Guangzhou, China)	Cat# CC9048
Oligonucleotides		
See Table S1 for qRT-PCR primers	This paper	N/A
Software and algorithms		
ZEN	Carl Zeiss	https://www.zeiss.co.jp/microscopy/ products/microscopesoftware/zen.html
ImageJ	NIH	https://imagej.nih.gov/ij/
ACDsee	ACD Systems International Inc	https://www.acdsee.cn
MetaboAnalyst 4.0	-	https://www.metaboanalyst.ca/MetaboAnalyst/faces/home.xhtml
R	MathSoft	https://www.r-project.org
Compound Discoverer 3.1 (CD 3.1) software	Thermo Fisher Scientific Inc.	-
GraphPad Prism 9	GraphPad Software	https://www.graphpad.com/
Other		
Glass slide	Citoglas	Cat# 7105 P
PVDF transfer membrane	Merck Millipore	Cat# GVWP02500

### Experimental model and subject details

#### Animals and experimental groups

All experimental procedures were approved by the Animal Ethics Committee of the University of South China (permit number: USC2020031602). 48 male C57BL/6 J mice (4 weeks, 16 ± 2 g) were obtained from the Laboratory Animal Center of the University of South China (Hengyang, China; permit number: SYXK (Xiang) 2020-0002). Free access to food and water, the mice were kept in a temperature-controlled environment (24 ± 2 °C) with a regular dark–light (12 h:12 h) cycle, which were randomly separated into control (Ctrl, *n* = 18) and experimental (OBE, *n* = 30) groups after a week of acclimatization. To establish the OBE model, the Ctrl group was given a normal diet (ND), whereas the OBE group was given a HFD rich in 60% fat for 12 weeks [82]. The mice which reached body weights of more than 120% of the mean body weight of the Ctrl group mice satisfied the criteria for an obese animal model [83]. The experimental group was then separated into an OBE group (*n* = 18) and an OBEPQQ group (*n* = 12) at random (This number of experimental animals in each group satisfied the modeling for serum metabolomics analysis, which was limited by the dose of drugs and HFD. Therefore, the sample size of serum metabolome was larger than that of other experiments). In the treatment experiment for 8 weeks, mice were divided into three groups as follows: Ctrl group, OBE group, and OBEPQQ group. The Ctrl and OBE mice were fed as described. The OBEPQQ mice were fed a HFD and intragastric administration of PQQ (10 mg/kg/d, dissolved in normal saline [68]).

#### Cell culture and transfection

TM3 cells were purchased from Cellcook Biotech Co., Ltd. and cultured in Dubecco’s Modified Eagle Medium Nutrient Mixture F-12 (DMEM/F-12) with 5% certified fetal bovine serum and 2.5% horse serum at 37 °C in a humid atmosphere with 5% CO_2_. The cells were first seeded in a 6 wells plate for the treatments and grown for 48 h. Subsequently, 0.4 mM palmitic acid (PA), 100 nM MCC950, and 100 nM PQQ were added to the medium for 24 h.

For cell transfection, about 0.4 mM PA-treated TM3 cells (2 × 10^5^/well) in a 6-well plate were transfected with 50 nM negative control and mouse-specific small interference RNA (siRNA) against PCSK9 synthesized by RiboBio, using a riboFECT CP Transfection Kit according to the manufacturer’s instructions. After transfection for 24 h at 37 °C, the culture medium was changed by a fresh complete culture medium. qRT-PCR and WB were conducted to analyze the transfection efficiency after transfection for 24 h. Then the cells were lysed and used for experimentation. The siRNA sequences were as follows (stB0001516A, RiboBio, China): genOFFTM st-h-PCSK9_001: (5′-GAGGTGTATCTCCTAGACA-3′).

The experimental groups and administration are as follows: Ctrl group (TM3 cells were treated with a fresh complete culture medium), NC group (TM3 cells were transfected with 50 nM negative control siRNA), siRNA group (TM3 cells were transfected with 50 nM siRNA against PCSK9). PA group (TM3 cells were treated with 0.4 mM PA), PA+siRNA group (0.4 mM PA-treated TM3 cells were transfected with 50 nM siRNAs against PCSK9), PA + MCC950 group (0.4 mM PA-treated TM3 cells were cultured with 100 nM MCC950), and PA + PQQ group (0.4 mM PA-treated TM3 cells were cultured with 100 nM PQQ).

### Method details

#### Oral glucose tolerance tests

The mice were gavaged with a bolus of glucose (2.0 g/kg body weight). Blood samples were collected from the tail at 0, 30, 60, and 120 min after the gavage.

#### Assessment of semen quality

The caudal epididymis was dissected and placed in 1.5 mL normal saline, which was cut up in order to give motility sperm time to escape into liquid, the caudal epididymis was left undisturbed for 20 min, heated to 37˚C. Then the 20 µl of sperm suspension was transferred into a Neubauer hemocytometer chamber for the assessment of the total number of sperm and Abnormal sperm rate with a light microscope (x 200) in at least 10 microscopic fields [84]. Sperm motility was estimated by counting the percentages of motile sperms in five separate and random fields each microscopic field with more than 200 sperms [85].

#### Morphological analysis of the liver, abdominal adipose, and testes

Finally, the mice were anesthetized with urethane (0.6 mL/100 g), and the liver, abdominal adipose, and testes were dissected and weighed to calculate the organ index (% of body weight). Lee’s index was calculated as follows: Lee’s index = (body weight)^1/3^ / body length × 10. The abdominal adipose and testes from different mice were fixed in 4% paraformaldehyde for 24 h and flushed with water for another 24 h. Then, the tissues were embedded in paraffin and sectioned into 5 μm-thick slices, which were stained with hematoxylin and eosin (H&E) for observing the morphology. Then, the diameters and area of 50 seminiferous tubules from each group were randomly evaluated by using an ocular micrometer with a light microscope (BX43; Olympus, PA, USA). Besides, Liver tissues were fixed in 4% PFA and gradually incubated in gradient sucrose solutions (30%, 50%, and 70% sucrose) for more than 12 hours. Then the tissue embedded in OCT compound and Oil Red O staining was performed on 8-m frozen sections. The differentiated mature adipocytes were rinsed in PBS and fixed with 4% PFA overnight before being treated for 10 minutes with a working Oil Red O solution (in 60% isopropanol) [86]. Oil red O staining of the liver was used as an effective method to assess liver steatosis on frozen sections [87]. The remaining tissues were quickly frozen in liquid nitrogen and stored at −80 °C.

#### Determination of the hormone levels of lipid metabolizing

Blood was collected from the abdominal aorta of mice when sacrificed and placed at room temperature (23 ± 2°C) for 30 min before centrifugation at 2500 rpm for 15 min at 4 °C. The levels of serum, TM3 cells and the supernatant of cells of Total Cholesterol (TC), Triglycerides (TG), LDL-C and high-density lipoprotein cholesterol (HDL-C) were measured using assay kits. In essence, the enzyme-coupled reactions resulted in the formation of quinone imide, which can be measured spectrophotometrically at 510 nm (TC or TG) and 546 nm (LDL-C or HDL-C). The concentration (mmol/L) of TC or TG in the assay sample was estimated from the equation: [(sample OD - control OD)/(standard OD - control OD)] × standard concentration (mmol/L). The concentration (mmol/L) of LDL-C or HDL-C in the assay sample was estimated from the equation: [(sample OD1 - sample OD1)-(control OD1- control OD1)]/[(sample OD2 - sample OD2)-(control OD2- control OD2)] × standard concentration (mmol/L).

#### Testosterone’s determination

The supernatant of serum was isolated for detecting T with radioimmunoassay by the Beijing Research Institute of Biotechnology of the North. The levels of T in cell culture supernatant were measured with an enzyme-linked immunosorbent assay (ELISA) kit according to the manufacturer’s instructions. The quantity of protein is measurable at 450 nm.

#### Transcriptome sequencing and bioinformatics analysis

Liver and testes RNA sequencing was carried out by Novogene (Beijing, China). In brief, to evaluate RNA purity and concentration, a NanoPhotometer® spectrophotometer (Implen Inc., CA, USA) and the Qubit® RNA Assay Kit in Qubit® 2.0 Fluorometer (Life Technologies, CA, USA) were used. Following the manufacturer’s instructions, a transcriptome sequencing library was created with 3 g RNA per sample using the NEBNext® UltraTM RNA Library Prep Kit for Illumina® (California, USA), as well as index codes were added to attribute sequences to each sample. The TruSeq PE Cluster Kit v3-cBot-HS (Illumina, California, USA) was used to cluster the index-coded samples on a cBot Cluster Generation System. Then the prepared library was sequenced on an Illumina Hiseq platform, yielding 150 bp paired-end reads. The DESeq2 R package (1.10.1) was used to perform differential expression analysis between various groups. DESeq2 adjusted p-values of gene for the sake of determining which genes were differentially expressed. The cluster Profiler R package was used to perform Gene Ontology (GO) enrichment analysis of differentially expressed genes (DEGs). Significantly enriched GO items were regard as the adjusted p-value of which less than 0.05. The cluster Profiler R program was performed to examine the statistical enrichment of DEGs using KEGG pathways (http://www.genome.jp/kegg/) [88].

#### LC–MS/MS analysis

All serum sample tubes were thawed at room temperature (RT), and then 100 µl of each sample was transferred into a new 1.5 ml centrifuge tube. The samples were vortexed for 15 s after the addition of 300 µl of acetonitrile. The mixture was then centrifuged (13,000 rpm/min, 4 °C) for 15 min. The supernatant fraction was collected and dried in vacuo. Vortexed it for 15 s after added 100 μL of 75% methanol solution to dissolve the dried metabolites, The mixture was then centrifuged (13,000 rpm/min, 4 °C) for 15 min, and the respective supernatants were transferred to a fresh vial for LC–MS (Thermo, Ultimate 3000LC, Q Exactive).

The LC–MS/MS analyses were carried out with a Dionex UltiMate 3000 UHPLC (Thermo Fisher Scientific Inc., Waltham, MA, USA) system with an ACQUITY UPLC BEH C18 column (50 mm × 2.1 mm, 1.7 μm, Techway, CHN) preheated to 30 °C. The mobile phase was composed of solvent A (aqueous 0.1% (v/v) formic acid) and solvent B (methanol) delivered at 0.30 mL/min with the following gradient: 0–4 min, 5% B, 4–20 min, 5% B–100% B, 20–23 min, 100% B, 23–25 min, 100% B–5% B. The injection volume was 3 µL.

The Q Exactive mass spectrometer (Thermo Fisher Scientific Inc., Waltham, MA, USA) was used to obtain sample mass spectrum data using positive ion or negative ion scan mode with spray voltage 3.0 kV and capillary temperature of 320 °C. The flow rate of sheath gas was 30 psi and aux gas was 10 arb. The full scan mode scanned from m/z 100–1000.

#### Immunohistochemical analysis (IHC) of testes

The antigen in 5 µm-thick dewaxed slices were retrieved by microwave treatment and the endogenous peroxidase was inactivated. Then, the sections were blocked with 5% bovine serum albumin for 45 min. Next, slices were incubated with polyclonal primary antibodies overnight at 4 °C. After washing with PBST (Phosphate-buffered saline containing 0.1% Tween 20), the slices were incubated with Rabbit Anti-Goat IgG(H + L), Biotin conjugate at room temperature and 37 °C for 45 min. Antibody bound to the section was visualized in DAB solution, and PBST was used as a negative control. Finally, the stained sections were observed and photographed under a light microscope (BX43, Olympus).

#### RNA isolation and quantitative real-time PCR (qRT-PCR)

Total RNA from testes tissues and TM3 cells was extracted using the TRIzol reagent and reversed to cDNA using TransScript® One-Step gDNA Removal and cDNA Synthesis SuperMix Kit according to the manufacturer’s protocol. The cDNA in same treatment groups were then randomly allocated into one experimental sample (each comprising 3 samples). Real-time PCR analyses were performed using the ChamQ Universal SYBR qPCR Master Mix with QuantStudio 7 Flex Real-Time PCR System (Thermo Fisher, Waltham, MA, USA). Primers were shown in Table S1. Data were normalized against endogenous GAPDH and quantification of the fold change was calculated using the 2^−ΔΔCt^ method. Experiments were performed at least three times.

#### Western blot (WB) analysis

Proteins were purified from testis tissues or TM3 cells. Western blot analyses were performed as detailed in a previous report [89]. Finally, eECL was added and Tanon-5500 Chemiluminescence Imaging System was used to detect chemiluminescence and protein bands. Band intensities were quantified using ImageJ software.

#### Cell cytotoxicity

To assess adherence to target cells and resulting cytotoxicity induced by different interventions, TM3 cells were plated in two 16 well plate overnight. On day 2, different interventions were added for further dynamic cell-growth monitoring via real-time cell analysis (RTCA). Cell Counting Kit-8 (CCK8) assay was implemented to analyze cell viability in TM3 cells treated by PA, MCC950 and PQQ in different concentrations. TM3 cells in 96-well plates were mixed with 20 μL CCK8 solution for 4 h. The absorbance (450 nm) was analyzed.

#### Pyroptosis detection

For the LDH release assay, the supernatants of TM3 cells were calculated for the presence of the cytoplasmic enzyme LDH using the LDH Cytotoxicity Assay Kit according to the manufacturer’s instructions. The percentage of cytotoxicity was evaluated as 100 × (experimental LDH - spontaneous LDH)/(maximum LDH release - spontaneous LDH). For the PI uptake assay, 5 μL Propidium iodide staining solution was added to the 1 mL 1 × Binding Buffer which was diluted by 10 × Binding Buffer. Then Cell cultures after treatment incubated in mentioned compound under light shielding. For the propidium iodide (PI) uptake assay, the fluorescence intensity of the retained PI was detected at an excitation wavelength of 536 nm [90].

### Quantification and statistical analysis

#### Data analysis of experiments

Data analysis and visualization were performed by GraphPad Prism v.8.0 (GraphPad Software, CA, USA) and presented as the mean ± standard deviation (SD). The student’s *t*-test was used to compare the data between two independent groups, whereas significant differences among multiple treatment comparison were analyzed using one-way ANOVA tests followed by Tukey’s test post hoc. Correlation arrays of transcriptome and hormone level was generated using Spearman’s rank correlation coefficients in the SPSS version 26.0 (SPSS Inc. Chicago, USA). *P*-value less than 0.05 was considered as statistical significance.

#### Metabolomics data analysis

Analyzed and processes the mass spectrum data by Compound Discoverer 3.1 (CD 3.1) software (Thermo Fisher Scientific Inc., Massachusetts, America). The *P*-values were obtained by Student’s *t*-test in CD 3.0 software. Multivariate statistical analyses including unsupervised principal component analysis (PCA) and orthogonal partial least squares discriminant analysis (OPLS-DA) were performed using the SIMCA-P 14.1 software package (Umetrics, Umea, Sweden). To further validate the OPLS-DA model, permutation tests (200 times) were performed. The variable importance (VIP) in the projection is obtained by OPLS-DA. Significantly different metabolites were screened using variable projected importance scores (VIP > 1) and P-values (*P* < 0.05). Metabolite pathway analysis was performed using MetaboAnalyst 4.0 (https://www.metaboanalyst.ca/MetaboAnalyst/faces/home.xhtml). The difference metabolite datasets in serum was visualized using heat maps (“pheatmap” package in R, https://www.r-project.org).

### Reporting summary

Further information on research design is available in the Nature Research Reporting Summary linked to this article.

### Supplementary information

Reporting Summary

Ethical Approval Documentation

Supplementary Figure 1

Supplementary Figure 2

Supplementary Figure 3

Supplementary Figure 4

Supplementary Figure 5

Supplementary Table 1

Supplementary Table 2

Supplementary Table 3

Supplementary Table 4

Supplementary Table 5

Original Data File

## Data Availability

All data reported in this paper will be shared by the lead contact upon request. This study did not generate custom code. Any additional information required to reanalyze the data reported in this paper is available from the lead contact upon request.

## Materials availability

This study did not generate new unique reagents.

## Code availability

All data reported in this paper will be shared by the lead contact upon request. This study did not generate custom code. Any additional information required to reanalyze the data reported in this paper is available from the lead contact upon request.
