# Supplementary material for: Pyrroloquinoline quinone inhibits PCSK9-NLRP3 mediated pyroptosis of Leydig cells in obese mice
Source: Cell Death Dis. 2023 Nov 7;14(11):723. doi: 10.1038/s41419-023-06162-8 (PMC10630350; doi:10.1038/s41419-023-06162-8)
Supplement: Supplementary file 1 — Reporting Summary [file 41419_2023_6162_MOESM1_ESM.docx]

**Corresponding Author Name:**__Xiaocan Lei_______________
**Manuscript Number:**_____CDDIS-23-1092R____________


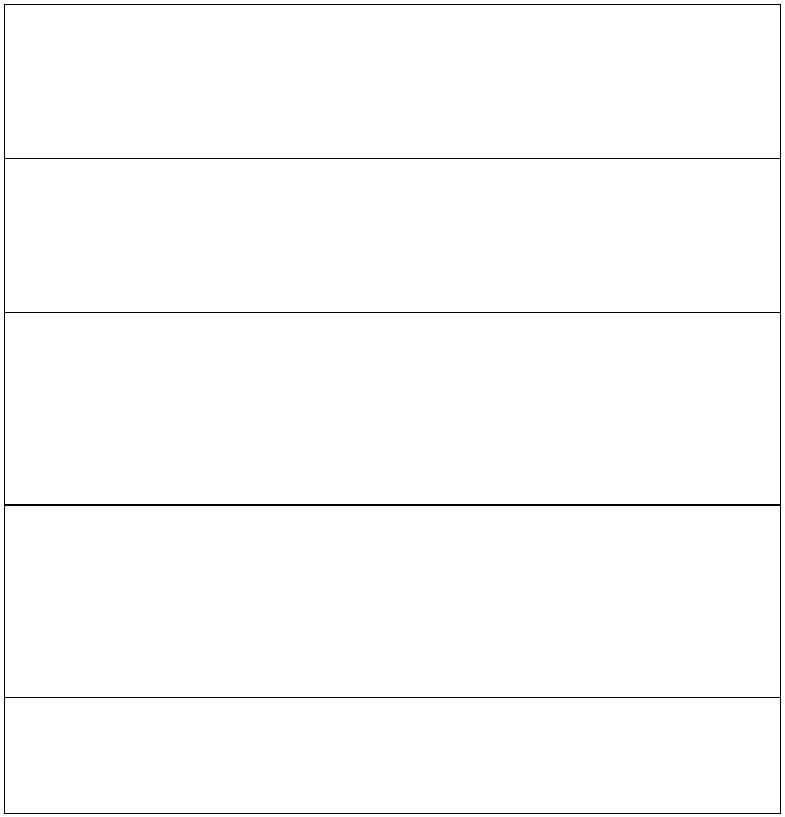

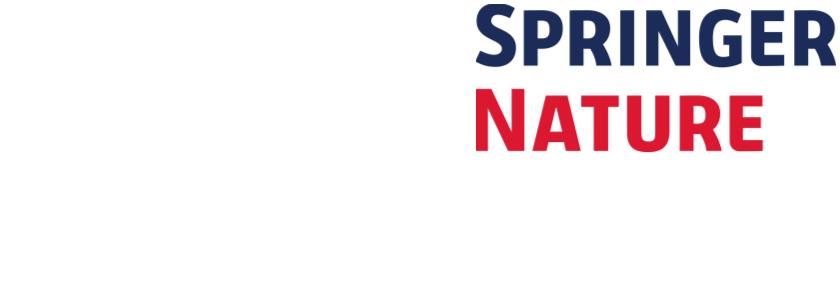

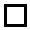


Reporting Summary

***Springer Nature wishes to improve the reproducibility of the work that we publish. This checklist is used to ensure good
reporting standards and to improve the reproducibility. Please respond completely to all questions relevant to your
manuscript. For more information, please read the journal’s Guide to Authors.***

☐ **Check here to confirm that the following information is available in the Material & Methods section:**

**The exact sample size (*n)*** for each experimental group/condition, given as a number, not a range
 **A description of the sample collection** allowing the reader to understand whether the samples represent
 technical or biological replicates (including how many animals, litters, culture, etc.)

**A statement of how many times the experiment shown was replicated in the laboratory** **Definitions of statistical methods and measures**: For small sample sizes (n<5) descriptive statistics are not
 appropriate, instead plot individual data points

o Very common tests, such as *t*-test, simple χ^2^ tests, Wilcoxon and Mann-Whitney tests, can be
 unambiguously identified by name only, but more complex techniques should be described in the
 methods section

o Are tests one-sided or two-sided?

o Are there adjustments for multiple comparisons?
o **Statistical test results**, e.g., ***P* values**

o Definition of **‘center values’** as **median or mean**;
 o Definition of **error bars as s.d. or s.e.m. or c.i.**

***Please ensure that the answers to the following questions are reported in the manuscript itself. We encourage you to
include a specific subsection in the methods section for statistics, reagents and animal models. Below, provide the
page number or section and paragraph number.***

Statistics and general methods **Reported in section/paragraph or page #**

1. How was the sample size chosen to ensure

adequate power to detect a pre-specified effect
size? (Give section/paragraph or page #)

For animal studies, include a statement about sample
 size estimate even if no statistical methods were Reported in page 19, line 592
 used.

2. Describe inclusion/exclusion criteria if samples or
 animals were excluded from the analysis. Were
 the criteria pre-established? (Give Reported in page 19, line 596

section/paragraph or page #)

3. If a method of randomization was used to
 determine how samples/animals were allocated
 to experimental groups and processed, describe
 it. (Give section/paragraph or page #)

For animal studies, include a statement about
 randomization even if no randomization was used. Reported in page 19, line 592

*1*

Academic Journals Reporting Checklist, November 2020

4. If the investigator was blinded to the group
 allocation during the experiment and/or when
 assessing the outcome, state the extent of
 blinding. (Give section/paragraph or page #)


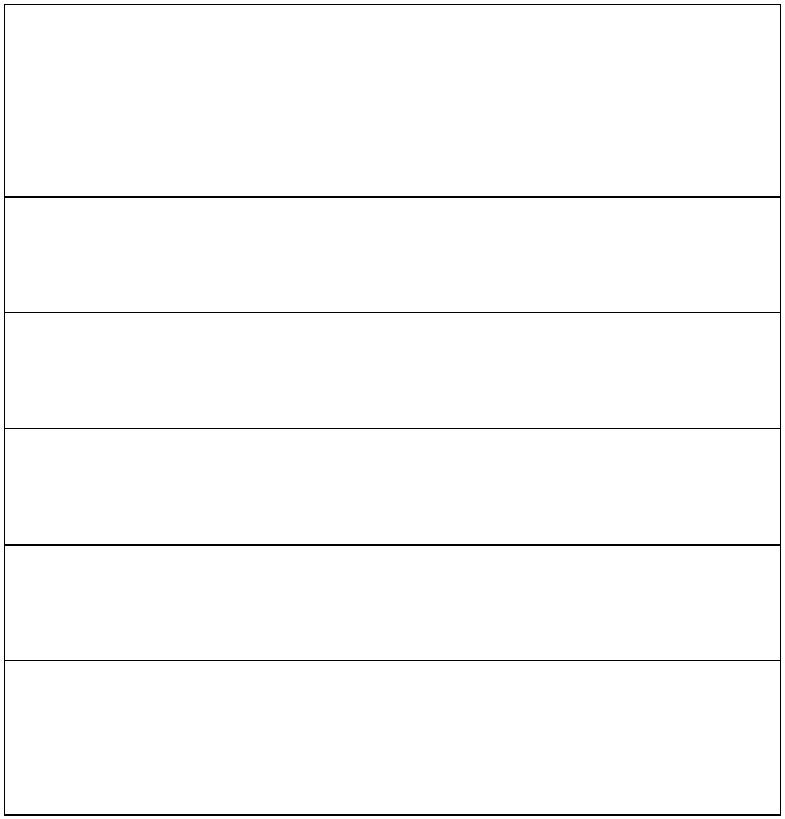

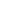


For animal studies, include a statement about
 blinding even if no blinding was done. Double-blindness was performed in this research.

5. For every figure, are statistical tests justified as
 appropriate? Yes.

Do the data meet the assumptions of the tests (e.g., The data meet the assumptions of the tests.
 normal distribution)?

Is there an estimate of variation within each group of
 data? Yes.

Is the variance similar between the groups that are Reported in page 25, line 776.
 being statistically compared? (Give
 section/paragraph or page #)

Reagents **Reported in section/paragraph or page #**

6. Report the source of antibodies (vendor and
 catalog number) Reported in Table 1.


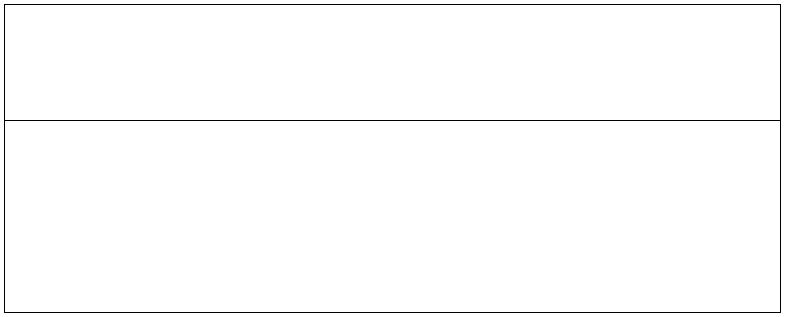


7. Identify the source of cell lines and report if they
 were recently authenticated (e.g., by STR
 profiling) and tested for mycoplasma
 contamination Reported in Table 1.

Animal Models **Reported in section/paragraph or page #**

8. Report species, strain, sex and age of animals


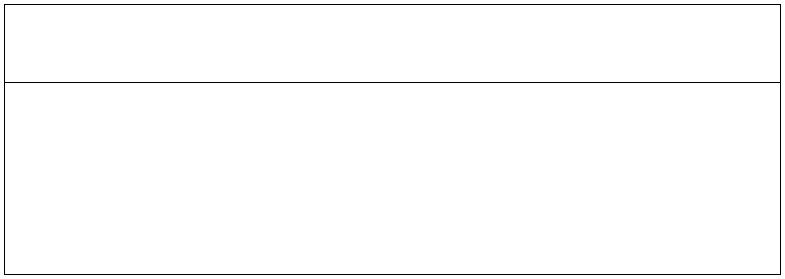


Reported in page 19, line 585.

9. For experiments involving live vertebrates,
 include a statement of compliance with ethical
 regulations and identify the committee(s)
 approving the experiments. Reported in Ethical Approval Documentation.

10. We recommend consulting the ARRIVE guidelines [(*PLoS Biol.* **8**(6), e1000412,2010) t](http://www.ncbi.nlm.nih.gov/pubmed/20613859)o ensure that other

relevant aspects of animal studies are adequately reported.

*2*

Academic Journals Reporting Checklist, November 2020

Human subjects **Reported in section/paragraph or page #**


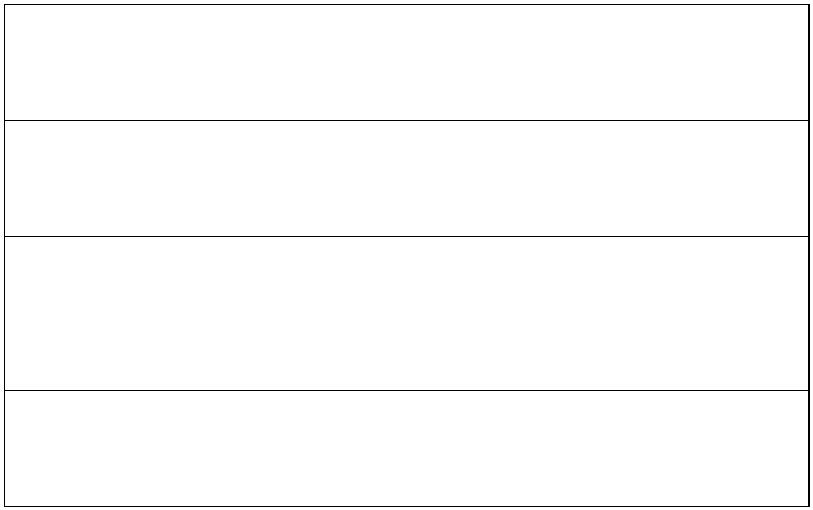

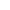

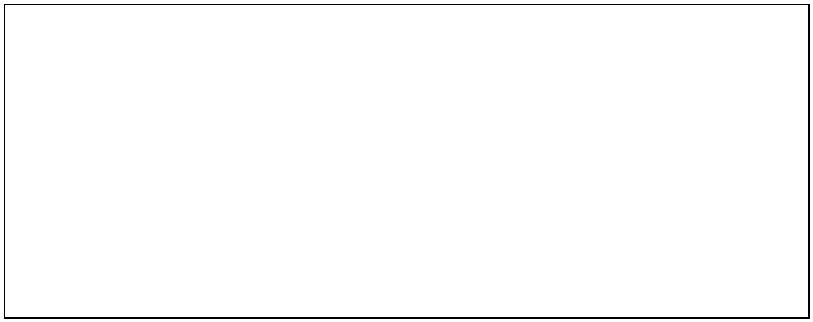

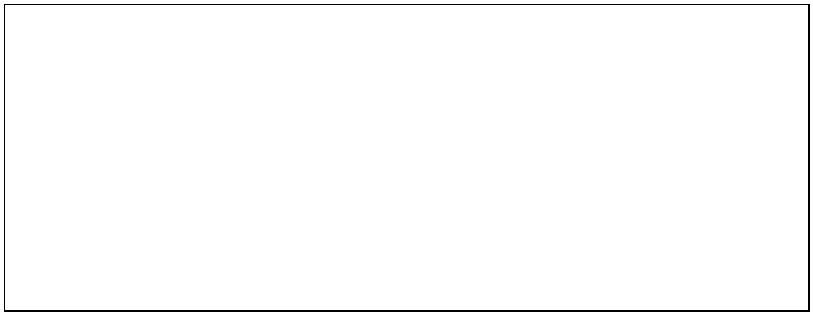


11. Identify the committee(s) approving the study
 protocol.

12. Include a statement confirming that informed
 consent was obtained from all subjects.

13. For publication of patient photos, include a
 statement confirming that consent to publish
 was obtained.

14. Report the clinical trial registration number (at
 [ClinicalTrials.gov](http://clinicaltrials.gov/) or equivalent).

15. For phase II and III randomized controlled trials, please refer to the [CONSORT statement](http://www.consort-statement.org/) and submit the
 CONSORT checklist with your submission.

16. For tumor marker prognostic studies, we recommend that you follow the [REMARK reporting guidelines.](http://www.nature.com/nrclinonc/journal/v2/n8/full/ncponc0252.html)

Data deposition **Reported in section/paragraph or page #**

17. Provide accession codes for deposited data.
 Data deposition in a public repository is
 Reported in page 19, line 580.

mandatory for:

a. Protein, DNA and RNA sequences
b. Macromolecular structures
c. Crystallographic data for small molecules
d. Microarray data

Deposition is strongly recommended for many other datasets for which structured public repositories exist; more
details on our data policy are available in the Guide to Authors. We encourage the provision of other source data
in supplementary information or in unstructured repositories such as [Figshare](http://www.figshare.com/) and [Dryad.](http://datadryad.org/) We encourage
publication of Data Descriptors (see [Scientific Data) t](http://www.nature.com/sdata/)o maximize data reuse.

18. If computer code was used to generate results
 that are central to the paper’s conclusions,
 include a statement in the Methods section
 under “**Code availability”** to indicate whether Reported in page 19, line 580.

and how the code can be accessed. Include
 version information as necessary and any
 restrictions on availability.

*3*

Academic Journals Reporting Checklist, November 2020
