# Supplementary material for: Pyrroloquinoline quinone inhibits PCSK9-NLRP3 mediated pyroptosis of Leydig cells in obese mice
Source: Cell Death Dis. 2023 Nov 7;14(11):723. doi: 10.1038/s41419-023-06162-8 (PMC10630350; doi:10.1038/s41419-023-06162-8)
Supplement: Supplementary file 2 — Ethical Approval Documentation [file 41419_2023_6162_MOESM2_ESM.pdf]

University of South China Application for Ethical Review of Laboratory Animal Welfare

Basic Information of Applicant					
**Project**	Effect of obesity and hyperglycemia on testicular spermatogenic disorders in male mice and its mechanism				
**Source of task**	horizontal subject		**Source of funding**		horizontal
**The experiment species:** mouse					
**Applicant**	Clinical Anatomy & Reproductive Medicine Application Institute, Heng Yang Medical College, University of South China				
**project leader**	Xiao-Can Lei	**telephone number**	17770910662	**E-mail**	2019000013@usc.edu.cn
**Contact Name**	Jin-Yuan Wang	**telephone number**	18554210179	**E-mail**	932510486@qq.com
**Objectives**	For the first time to apply for	**Authorization number** NO: 2021USA0628			

Basic information of experimental animals		
**specie:** mouse	**Breed:**C57BL/6J	
**Experimental animal qualification certificate number** NO: USC2020031602		
**Weight（g）：** 18	**Age（d）：** 28	**Class：** clean animal
**Gender：** male	**Quantity:**150	
**Productive establishment：** Hunan SJA Laboratory Animal limited company	**Production license number of experimental animals ：** SCXK(湘)2019-0004	
**Experiment site:** Department of animal	**umber of the license for the use of experimental animals ：** SYXK(湘)2020-0002	

Purpose of this study and its contribution to humans, animals or science：

Objective:

1. To explore the effects of obesity on male fertility and reproductive dysfunction and its molecular mechanism; 2. To explore the effects and molecular mechanisms of hyperglycemia on male fertility and reproductive dysfunction;
3. Explain the effect of nicotinamide mononucleotide and other drugs on the improvement and protection of reproductive dysfunction induced by obesity and hyperglycemia.

Contributions to human beings, animals or science:

To identify the main causes of the significant increase in male infertility rate from the perspective of animal models, and to develop more effective and most effective molecular targets for the treatment of obesity and diabetes-induced reproductive dysfunction, and to provide theoretical support for the research and development of new drugs.

Please focus on the "3R" principle of experimental animals and explain the necessity of animal experiments:

Several relevant domestic and foreign literatures have reported that C57BL6J strain mice are the best choice for the construction of animal models of diabetes and obesity. Our research group has conducted a large number of preliminary experimental studies in the early stage, and the results show that the animal models of diseases that meet the clinical reports can be successfully constructed. In addition, this research group conducted a long-term analysis of the preventive and therapeutic effects of nicotinamide mononucleotide, spermidine, icariin, dendrobium polysaccharide, puerarin and other drug components on the disease model. According to the experimental design of relevant studies, about 150 mice were needed to carry out the test.

Design of animal experiments:

Drug administration program: Intragastric intervention was mainly carried out with harmless drugs reported in the study. The operation of intragastric administration was carried out with special injection for mice to relieve the pain of mice.

The animal marking number is marked by the tail of the marker pen, which does no harm to the mice.

The animal's guarantee is that the experimental operator picks up the mouse's back skin with his left hand and places it in the palm to fix it, which does no harm to the mice.

Euthanasia: At the end of the experiment, the specimens were collected and the mice were anesthetized. The blood, testis, epididymis, liver, kidney and other organs of the mice were collected under anesthesia for analysis, and the mouse model was fully utilized to carry out relevant experimental studies, so as to ensure the full utilization value and contribution of the mice.

Anticipation of pain in the experimental animal and pain relief measures:

During the pre-experiment, high-fat-diet with 60% fat, 10 mg/kg PQQ, 500 mg/kg NMN and 1ml syringes were used for intraperitoneal injection of mice. The size was suitable for the body of mice and the pain was greatly reduced. In the process of sampling, 0.6mg/kg Ulatan injection was used to anesthetize the mice, and the samples were collected in painless state.

Commitment

Commitment from the project leader

I hereby certify that the contents of this application form are accurate and correct.

I promise to strictly abide by the provisions of the Regulations on the Management of Laboratory Animals, the Guiding Opinions on the Treatment of Laboratory Animals, and the Measures of Hunan Province for the Management of Laboratory Animals.

I promise myself included the application of contact with experimental animals is described in table personnel, have participated in the south China university laboratory animal center for related training, to master the application of the animal experimental methods involved in the table, have the ability to carry out the animal experiment, use these animals and animal tissue and know the risks.

I know as head of the project, it is the responsibility of commitment to all members of this team in this research work will follow the principle of humanitarian, ensure laboratory animal welfare ethics, and strictly abide by the laboratory animal center at the university of south China related rules and regulations, accept the experimental animal welfare ethical review committee of the guidance and supervision and inspection.

Signature of project leader: Xiao-Can Lei

Xiao-Can Lei.

2020. 10. 1

The ethics committee will examine and approve opinions ：

Signature (seal) of the Presiding Committee:

南[illegible]大学医学伦理委员会

4304079008946

2020. 10. 5
