# Supplementary material for: Pyrroloquinoline quinone inhibits PCSK9-NLRP3 mediated pyroptosis of Leydig cells in obese mice
Source: Cell Death Dis. 2023 Nov 7;14(11):723. doi: 10.1038/s41419-023-06162-8 (PMC10630350; doi:10.1038/s41419-023-06162-8)
Supplement: Supplementary file 3 — Supplementary Figure 1 [file 41419_2023_6162_MOESM3_ESM.docx]

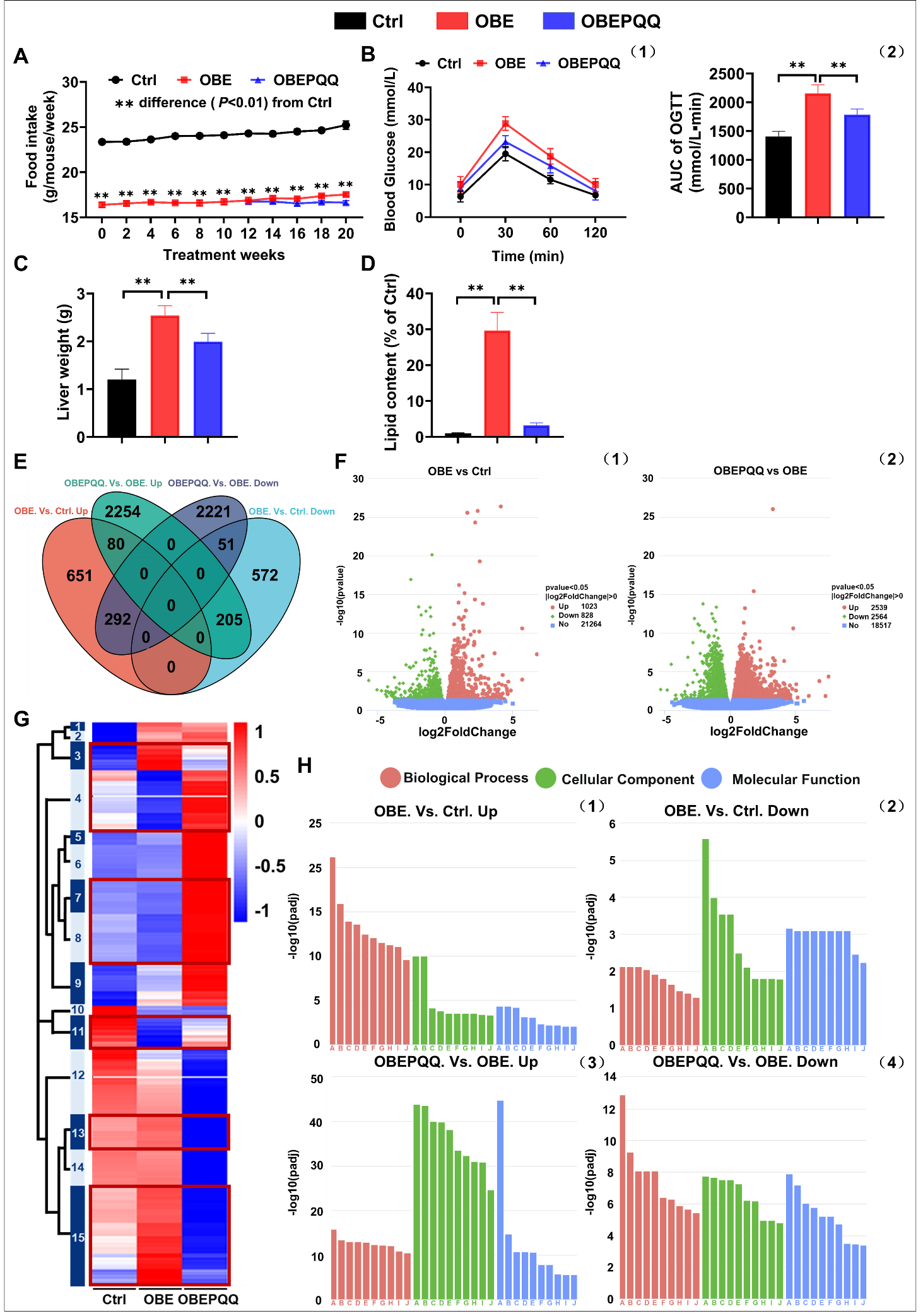


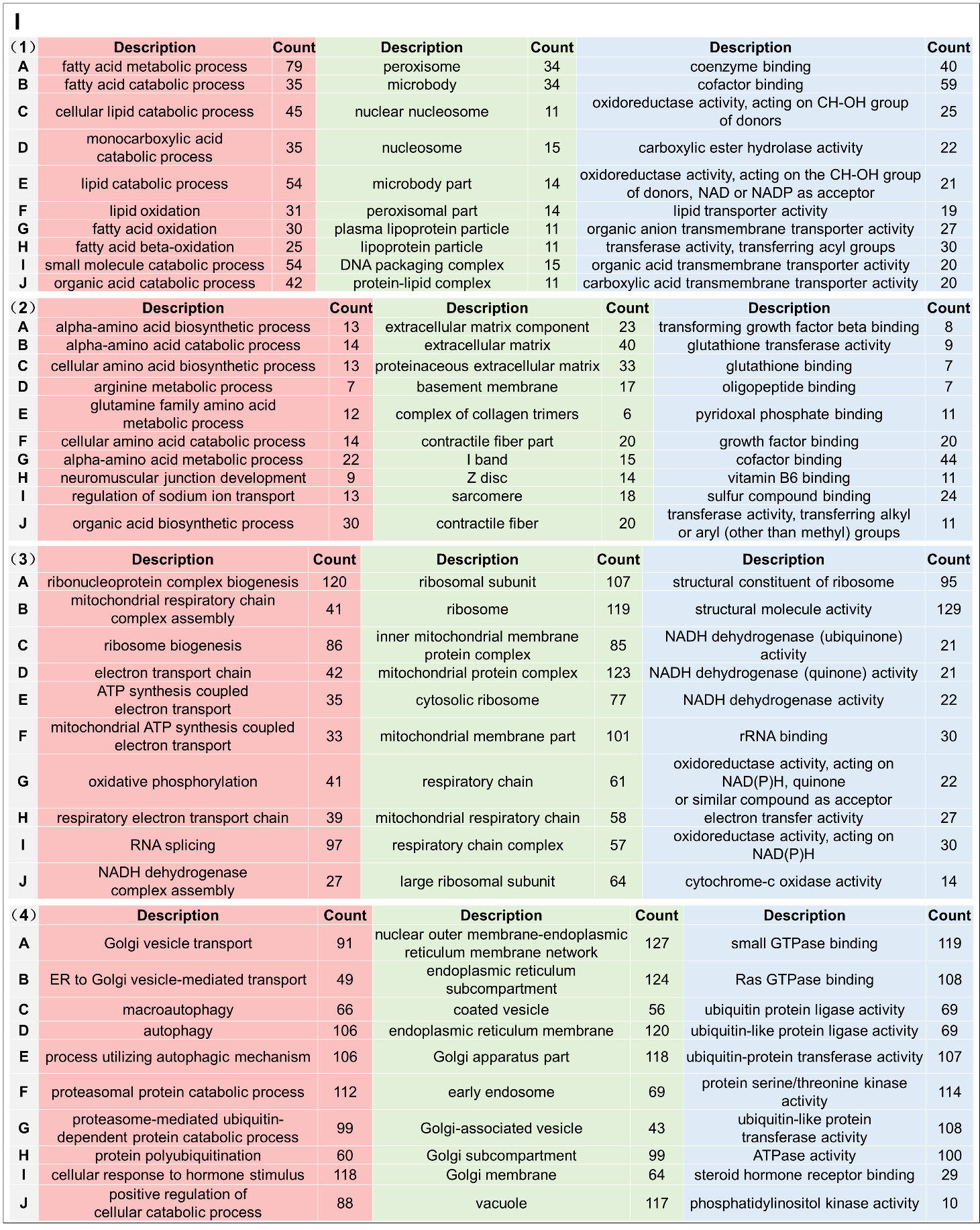


**Supplementary Figure 1. Transcriptomic profiling across the obesity reveals gene signatures for abnormal lipid metabolism in the liver.**

(A) Food intake of mice (n=10).

(B) OGTT in the three groups (1) and AUC of OGTT curves (n = 6).

(C) Liver weight (n=10).

(D) The quantification of Oil red levels in liver (n=8).

(E) Venn diagram of DEGs in the three groups (n=4).

(F) Volcano plots of liver’s DEGs in OBE vs. Ctrl groups (1) and OBEPQQ vs. OBE groups (2) (n=4).

(G) Heatmap presenting data from a microarray experiment performed with liver samples (n=4/group). Hierarchical clustering is also shown, which allows the definition of 15 gene clusters (p ≤ 0.05).

(H) The top 30 GO items enriched to the key GO terms.

(I) The details of Supplementary Figure 1H.
