# Supplementary material for: Pyrroloquinoline quinone inhibits PCSK9-NLRP3 mediated pyroptosis of Leydig cells in obese mice
Source: Cell Death Dis. 2023 Nov 7;14(11):723. doi: 10.1038/s41419-023-06162-8 (PMC10630350; doi:10.1038/s41419-023-06162-8)
Supplement: Supplementary file 4 — Supplementary Figure 2 [file 41419_2023_6162_MOESM4_ESM.docx]

**
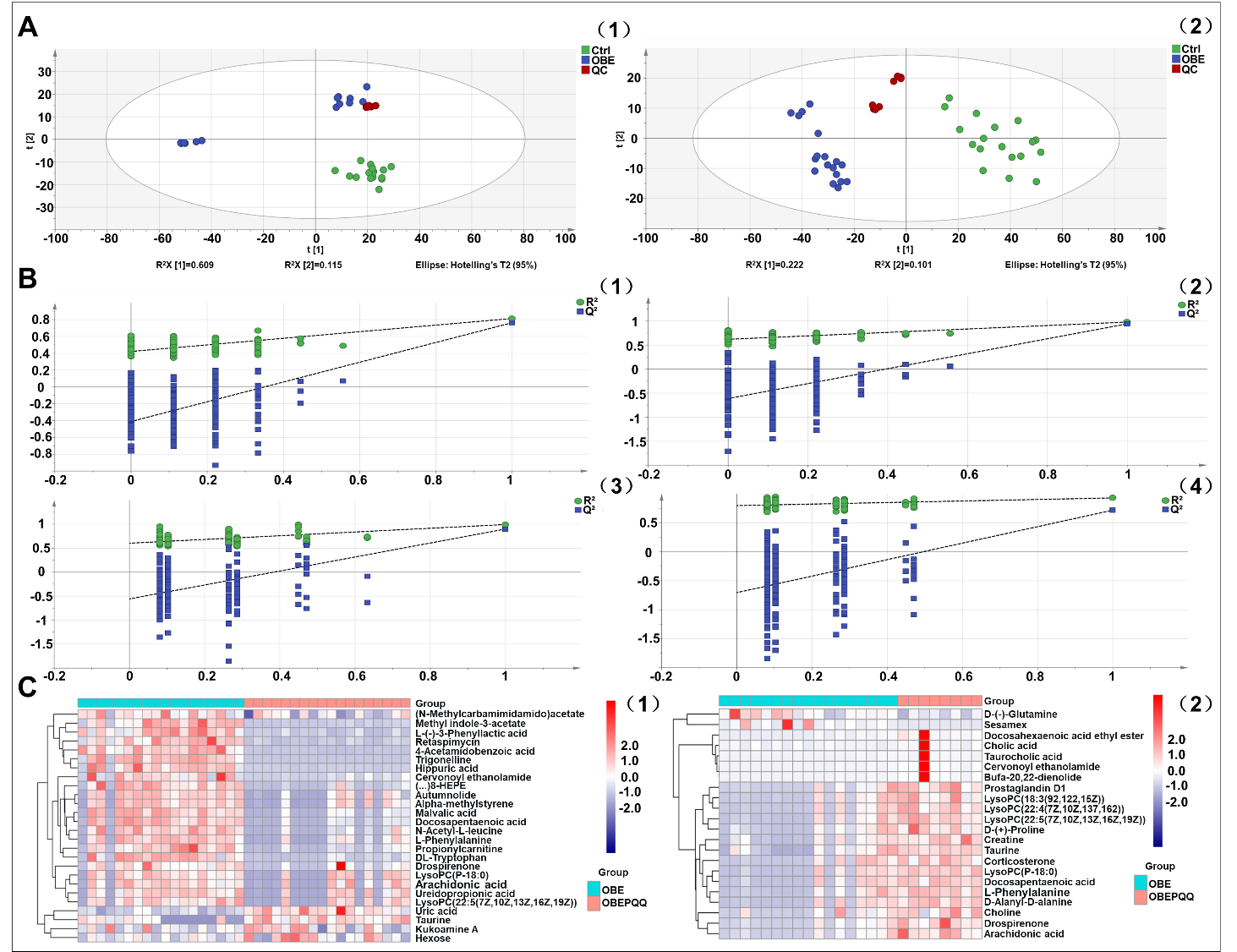
**

**Supplementary Figure 2.** **PQQ administration attenuates the abnormal cholesterol metabolism in obese mice.**

(A) PCA score plots of Ctrl and OBE group in the positive ion mode (1) and the negative ion mode (2), QC (Quality Control) samples reflect instrument stability (Ctrl, n=18; OBE, n=18; OBEPQQ, n=8).

(B) Permutation test for OPLS-DA in positive and negative ion mode between OBE and Ctrl group (1,2) and between OBE and OBEPQQ group (3,4).

(C) Heat maps of differential metabolites between Ctrl and OBE group (1) and between OBE and OBEPQQ group (2).
