# Supplementary material for: Pyrroloquinoline quinone inhibits PCSK9-NLRP3 mediated pyroptosis of Leydig cells in obese mice
Source: Cell Death Dis. 2023 Nov 7;14(11):723. doi: 10.1038/s41419-023-06162-8 (PMC10630350; doi:10.1038/s41419-023-06162-8)
Supplement: Supplementary file 5 — Supplementary Figure 3 [file 41419_2023_6162_MOESM5_ESM.docx]

**
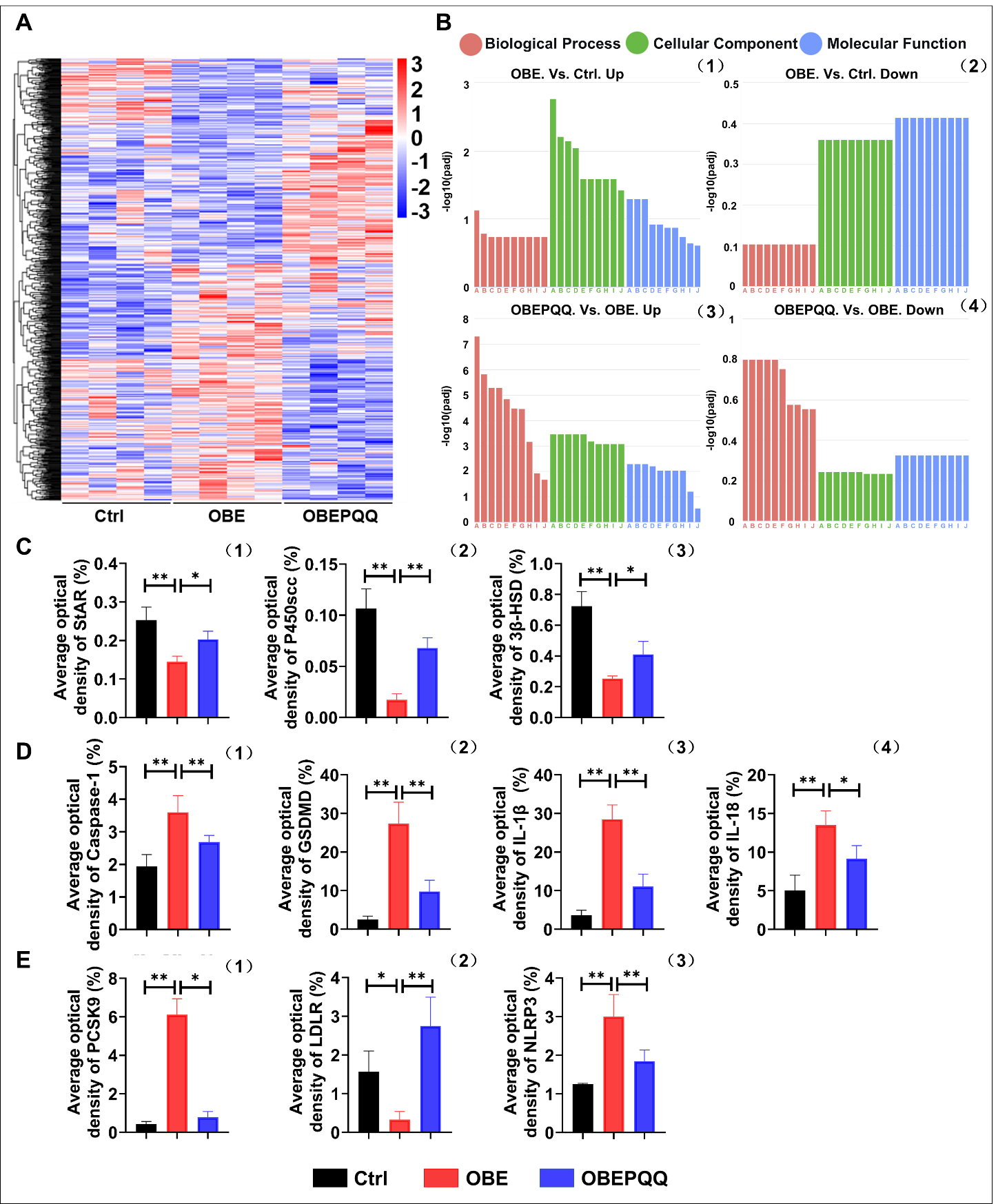
**

**
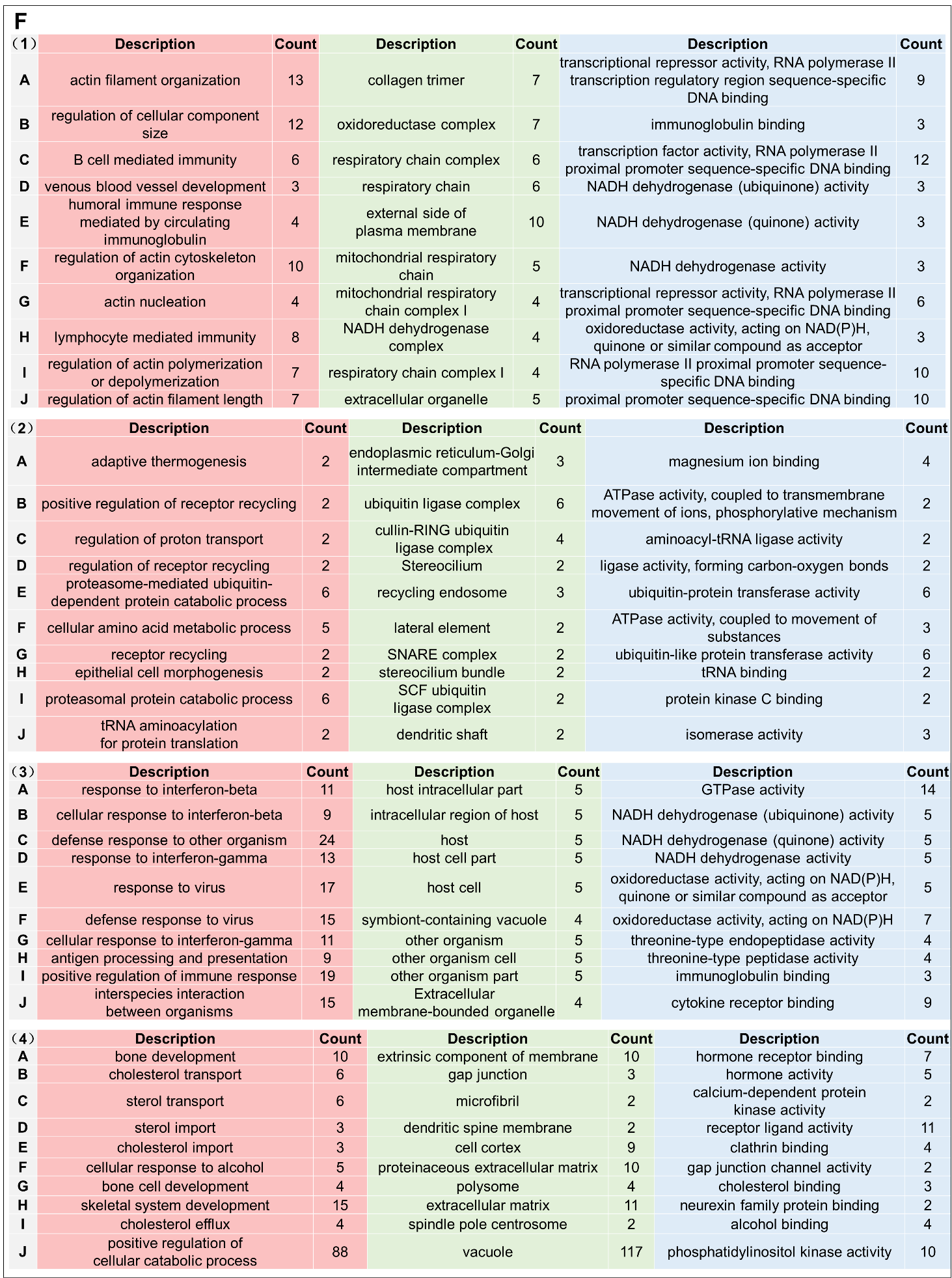
**

**Supplementary Figure 3.** **Transcriptomic profiling across obesity reveals gene signatures for abnormal lipid metabolism in the testes.**

(A) Heatmap presenting data from DEGs with testicular samples (n=4).

(B) The top 30 GO items enriched to the key GO terms.

(C) The quantification on the IHC stainings for the Figure 4H.

(D) The quantification on the IHC stainings for the Figure 5C.

(E) The quantification on the IHC stainings for the Figure 6C.

(F) The details of Supplementary Figure 3B.
