# Supplementary material for: Pyrroloquinoline quinone inhibits PCSK9-NLRP3 mediated pyroptosis of Leydig cells in obese mice
Source: Cell Death Dis. 2023 Nov 7;14(11):723. doi: 10.1038/s41419-023-06162-8 (PMC10630350; doi:10.1038/s41419-023-06162-8)
Supplement: Supplementary file 6 — Supplementary Figure 4 [file 41419_2023_6162_MOESM6_ESM.docx]

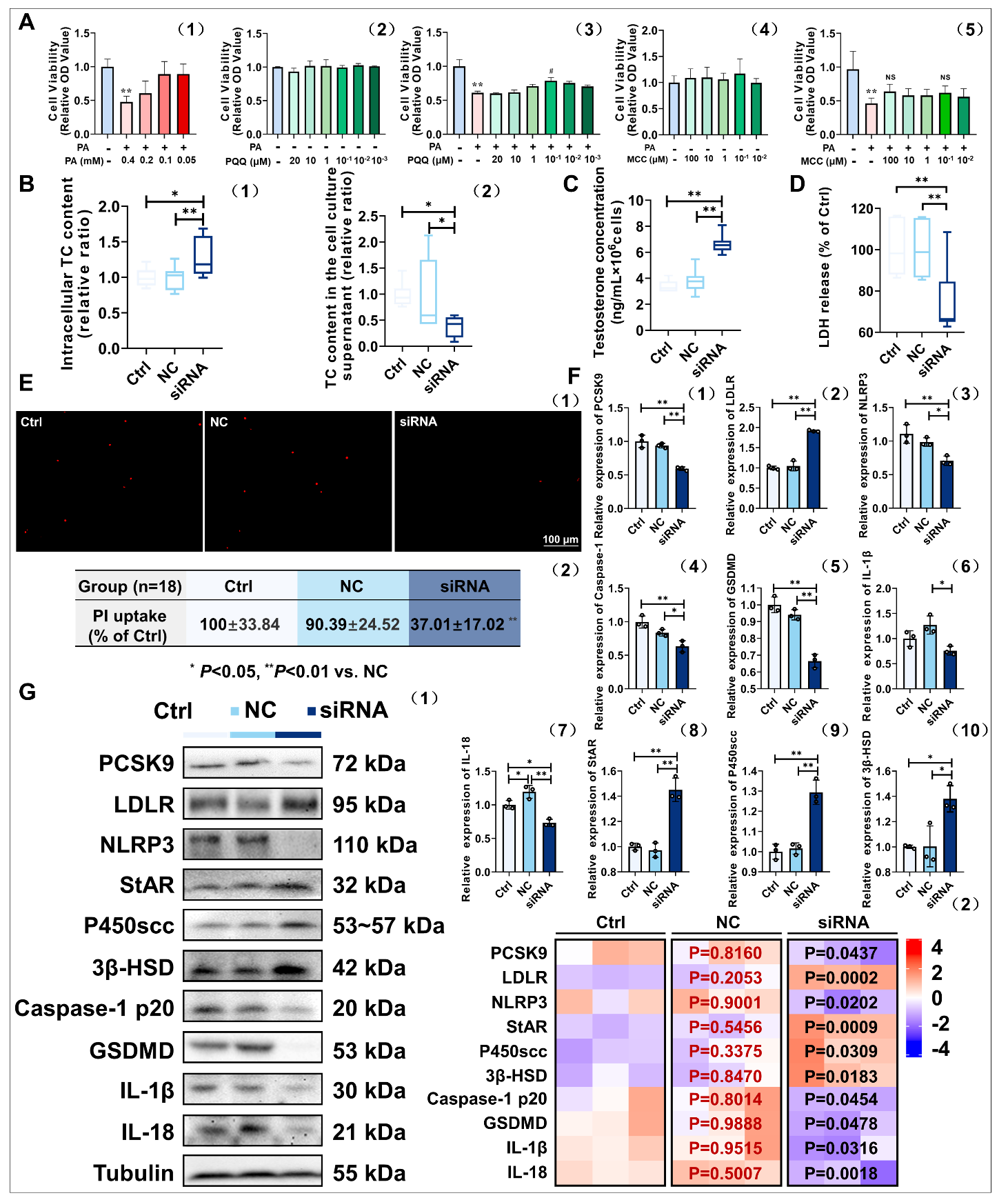


**Supplementary Figure 4. PCSK9 silencing ameliorates testosterone synthesis in Leydig cells.**

(A) Cell counting kit-8 (CCK-8) assay was performed to examine the cell viability, in order to assess the optimum concentration of PA (1), PQQ toxicity (2), the optimum concentration of PQQ (3), MCC toxicity (4) and optimum concentration of MCC (5) (n=3). ns means p > 0.05 vs Ctrl.

**(**B) Cholesterol levels in intracellular (1) (n=9) and TM3 cells culture supernatant (2) (n=8).

(C) The levels of testosterone synthesized by TM3 cells (n=8).

(D) LDH release was assayed by the LDH Cytotoxicity Assay Kit (n=10).

(E) The fluorescence intensity of PI was detected by a microplate absorbance reader (1) and statistical analysis (2).

(F) qRT-PCR validation of PCSK9, LDLR, NLRP3, StAR, P450scc, 3β-HSD, Caspase-1, GSDMD, IL-1β and IL-18 (n=3).

(G) Expression of PCSK9, LDLR, NLRP3, StAR, P450scc, 3β-HSD, Caspase-1 p20, GSDMD, IL-1β and IL-18 detected by WB analysis (1), and heatmap of that relative quantified analysis performed by ImageJ software (2). Relative quantitation of protein level normalized to tubulin (n=3); The red one indicates p-value vs Ctrl group, while the black one indicates p-value vs NA group. *p < 0.05, **p < 0.01.
