# Supplementary material for: Pyrroloquinoline quinone inhibits PCSK9-NLRP3 mediated pyroptosis of Leydig cells in obese mice
Source: Cell Death Dis. 2023 Nov 7;14(11):723. doi: 10.1038/s41419-023-06162-8 (PMC10630350; doi:10.1038/s41419-023-06162-8)
Supplement: Supplementary file 7 — Supplementary Figure 5 [file 41419_2023_6162_MOESM7_ESM.docx]

**
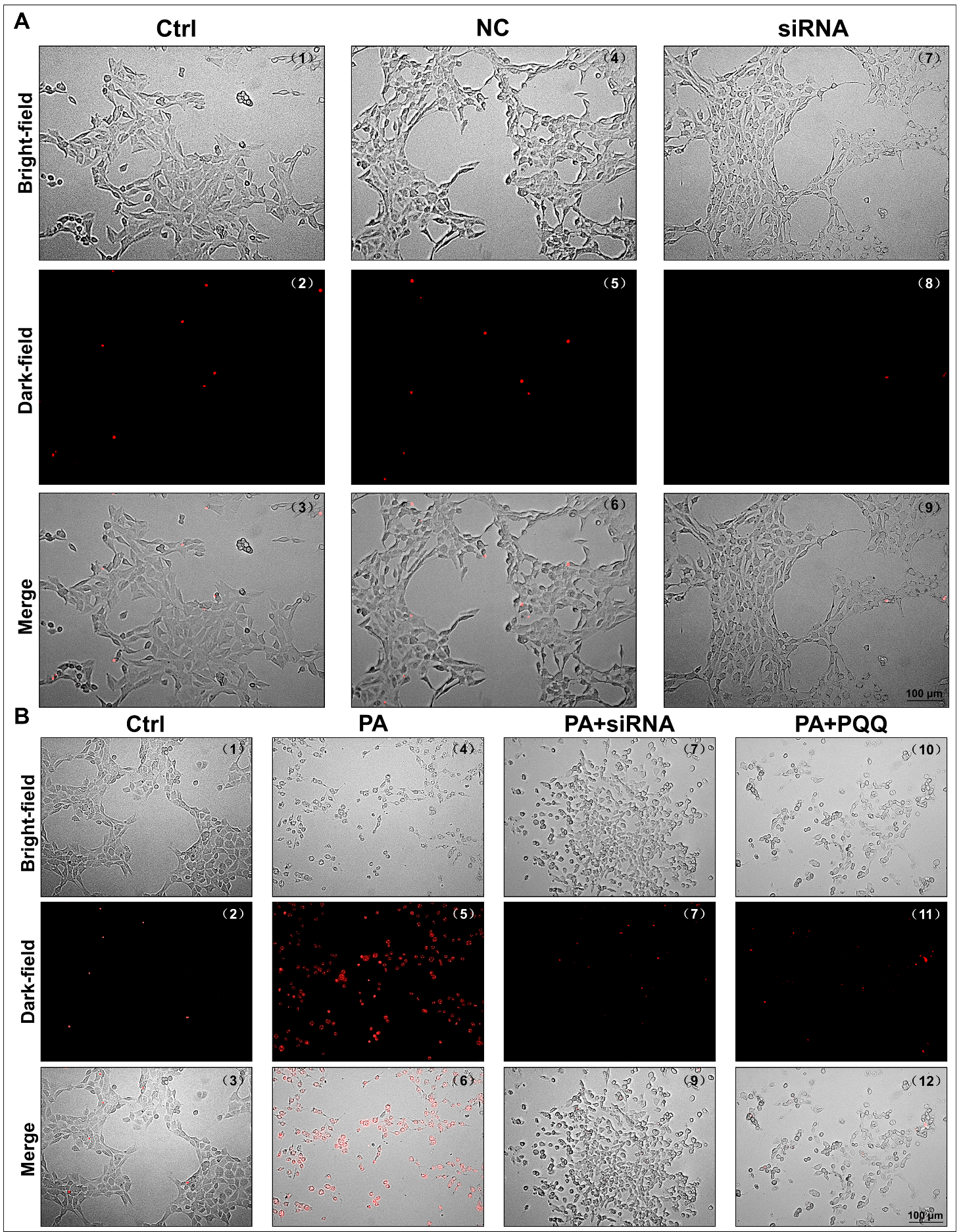
**

**
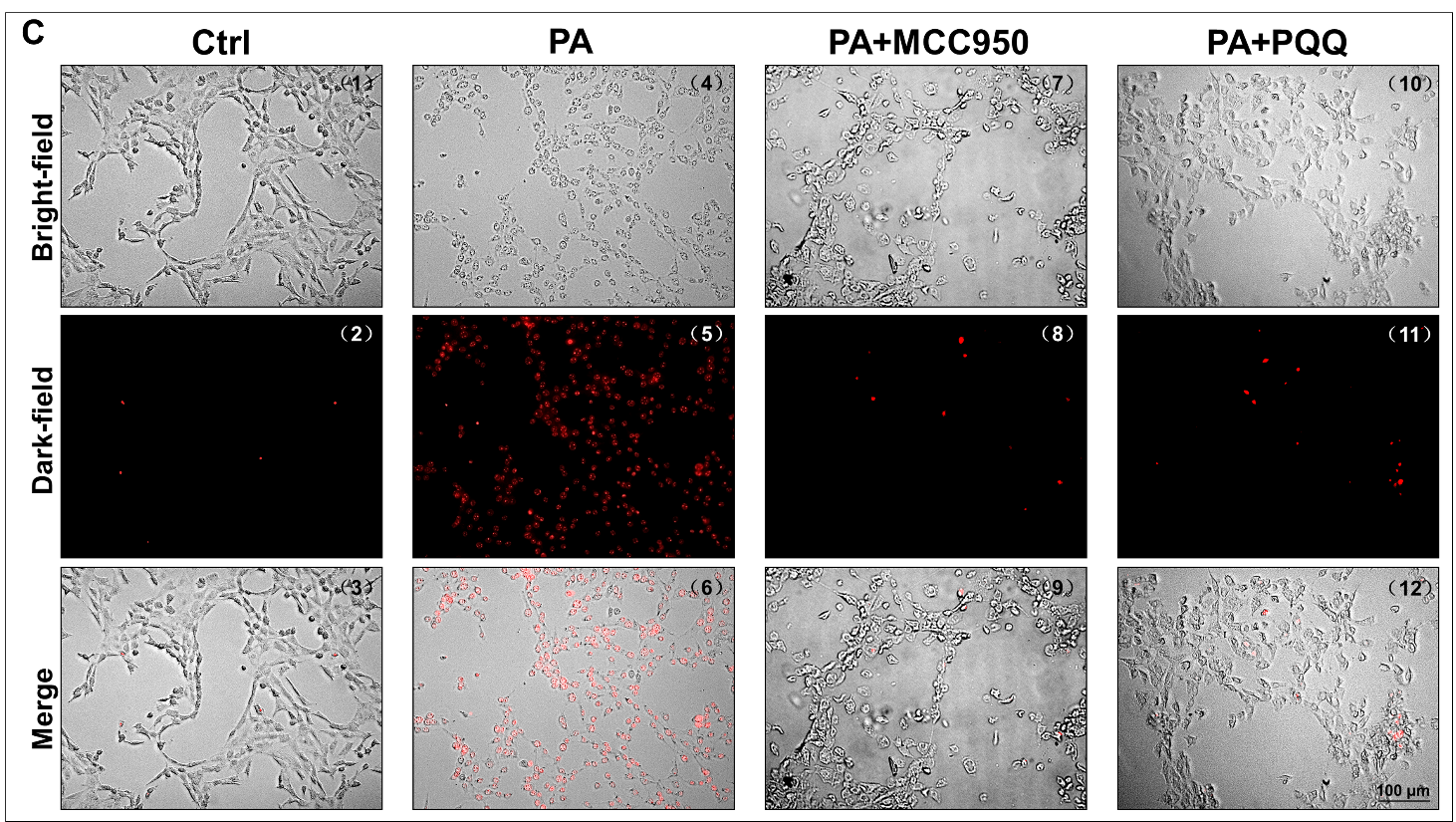
**

**Supplementary Figure 5. PCSK9 silencing and NLRP3 suppression ameliorates pyroptosis in Leydig cells.**

(A-C) The fluorescence intensity of PI (red) was detected by a microplate absorbance reader. Merged images could be constructed for the sake of evaluating pyroptosis condition in each treatment group.
