# Supplementary material for: Pyrroloquinoline quinone inhibits PCSK9-NLRP3 mediated pyroptosis of Leydig cells in obese mice
Source: Cell Death Dis. 2023 Nov 7;14(11):723. doi: 10.1038/s41419-023-06162-8 (PMC10630350; doi:10.1038/s41419-023-06162-8)
Supplement: Supplementary file 8 — Supplementary Table 1 [file 41419_2023_6162_MOESM8_ESM.docx]

**Table S1. Primer sequences of genes for quantitative qRT-PCR.**

| Gene | Sequence of forward and reverse primers 5'‑3' | NCBI reference sequenc |
| --- | --- | --- |
| PCSK9 | F: AGCAGCCAGGTGGAGGTGTATC  R: CTTGCTCGCCTGTCTGTGGAAG | NM_153565.2 |
| LDLR | F: GAGGAACTGGCGGCTGAA  R: GTGCTGGATGGGGAGGTCT | NM 001252659.1 |
| NLRP3 | F: ATCAACAGGCGAGACCTCTG  R: GTCCTCCTGGCATACCATAGA | NM_145827.4 |
| Caspase-1 | F: AGAGGATTTCTTAACGGATGCA  R: TCACAAGACCAGGCATATTCTT | NM_009807.2 |
| GSDMD | F: CGATGGGAACATTCAGGGCAGAG  R: ACACATTCATGGAGGCACTGGAAC | NM_026960.4 |
| IL-1β | F: ACCTTCCAGGATGAGGACATGA  R: GATTCTTTCCTTTGAGGCCCA | NM_008361.4 |
| IL-18 | F: CAGACCACTTTGGCAGAC  R: GATTTATCCCCATTTTCAT | NM_008360.2 |
| StAR | F: ATGTTCCTCGCTACGTTCAAG  R: CCCAGTGCTCTCCAGTTGAG | NM_011485.5 |
| 3β-HSD | F: AGCTCTGGACAAAGTATTCCGA  R: GCCTCCAATAGGTTCTGGGT | NM_008293.4 |
| P450scc | F: AGGTCCTTCAATGAGATCCCTT  R: TCCCTGTAAATGGGGCCATAC | NM_019779.4 |
| GAPDH | F: ATTGTCAGCAATGCATCCTG  R: ATGGACTGTGGTCATGAGCC | NM_001289726.1 |
