# Supplementary material for: Pyrroloquinoline quinone inhibits PCSK9-NLRP3 mediated pyroptosis of Leydig cells in obese mice
Source: Cell Death Dis. 2023 Nov 7;14(11):723. doi: 10.1038/s41419-023-06162-8 (PMC10630350; doi:10.1038/s41419-023-06162-8)
Supplement: Supplementary file 9 — Supplementary Table 2 [file 41419_2023_6162_MOESM9_ESM.docx]

**Table S2. NAFLD activity scoring of liver tissues.**

|  | Ctrl | OBE | OBEPQQ |
| --- | --- | --- | --- |
| Steatosis | 0 | 2.125±0.641** | 1.250±0.707^#^ |
| Lobular Inflammation | 0 | 1.000±0.756** | 0.125±0.354^#^ |
| Hepatocyte Ballooning | 0 | 0 | 0 |
| Total | 0 | 3.125±0.991** | 1.375±0.744^##^ |
| Fibrosis | 0 | 0 | 0 |

The total NAS score represents the sum of scores for steatosis, lobular inflammation, and ballooning and ranges from 0-8. Total scores of 0-2 was considered not NASH; 3-4 was evenly divided among those considered not NASH, borderline, or positive for NASH; 5-8 considered NASH. The data were represented as mean ± SD, n = 8, ***P* <0.01 vs Ctrl; ^##^*P* <0.01, ^#^*P* < 0.05 vs OBE.
