# Supplementary material for: Pyrroloquinoline quinone inhibits PCSK9-NLRP3 mediated pyroptosis of Leydig cells in obese mice
Source: Cell Death Dis. 2023 Nov 7;14(11):723. doi: 10.1038/s41419-023-06162-8 (PMC10630350; doi:10.1038/s41419-023-06162-8)
Supplement: Supplementary file 10 — Supplementary Table 3 [file 41419_2023_6162_MOESM10_ESM.docx]

**Table S3. Related parameters of the serum metabolomics analysis**.

| Index  Groups | PCA score plots | | OPLS-DA plots | | Permutation test | |
| --- | --- | --- | --- | --- | --- | --- |
|  | R^2^X | Q^2^ | R^2^Y | Q^2^ | R^2^ | Q^2^ |
| OBE vs Ctrl_p | 0.742 | 0.703 | 0.814 | 0.764 | 0.0, 0.429 | 0.0, -0.422 |
| OBE vs Ctrl_n | 0.646 | 0.295 | 0.979 | 0.942 | 0.0, 0.62 | 0.0, - 0.612 |
| OBEPQQ vs OBE_p | 0.877 | 0.685 | 0.993 | 0.889 | 0.0, 0.604 | 0.0, -0.559 |
| OBEPQQ vs OBE_n | 0.632 | 0.223 | 0.931 | 0.72 | 0.0, 0.801 | 0.0, -0.66 |
