# Supplementary material for: Pyrroloquinoline quinone inhibits PCSK9-NLRP3 mediated pyroptosis of Leydig cells in obese mice
Source: Cell Death Dis. 2023 Nov 7;14(11):723. doi: 10.1038/s41419-023-06162-8 (PMC10630350; doi:10.1038/s41419-023-06162-8)
Supplement: Supplementary file 11 — Supplementary Table 4 [file 41419_2023_6162_MOESM11_ESM.docx]

**Table S4. Related parameters of the differential metabolites identified pathways in serum metabolomics analysis (Ctrl vs OBE).**

| Index  Pathway | Total | Expected | Hits | Raw p | -log10(p) | Holm adjust | FDR | Impact |
| --- | --- | --- | --- | --- | --- | --- | --- | --- |
| Phenylalanine metabolism | 12 | 0.15936 | 2 | 0.010215 | 1.9907 | 0.8581 | 0.8581 | 0.35714 |
| Phenylalanine, tyrosine and tryptophan biosynthesis | 4 | 0.053121 | 1 | 0.052123 | 1.283 | 1 | 1 | 0.5 |
| Arachidonic acid metabolism | 36 | 0.47809 | 2 | 0.080639 | 1.0935 | 1 | 1 | 0.35412 |
| Taurine and hypotaurine metabolism | 8 | 0.10624 | 1 | 0.10166 | 0.99286 | 1 | 1 | 0.42857 |
| Pantothenate and CoA biosynthesis | 19 | 0.25232 | 1 | 0.22551 | 0.64683 | 1 | 1 | 0.02857 |
| beta-Alanine metabolism | 21 | 0.27888 | 1 | 0.24621 | 0.60869 | 1 | 1 | 0.10448 |
| Galactose metabolism | 27 | 0.35857 | 1 | 0.30521 | 0.5154 | 1 | 1 | 0.05288 |
| Glycine, serine and threonine metabolism | 34 | 0.45153 | 1 | 0.36849 | 0.43357 | 1 | 1 | 0 |
| Biosynthesis of unsaturated fatty acids | 36 | 0.47809 | 1 | 0.38554 | 0.41393 | 1 | 1 | 0 |
| Glycerophospholipid metabolism | 36 | 0.47809 | 1 | 0.38554 | 0.41393 | 1 | 1 | 0.01736 |
| Amino sugar and nucleotide sugar metabolism | 37 | 0.49137 | 1 | 0.3939 | 0.40461 | 1 | 1 | 0 |
| Arginine and proline metabolism | 38 | 0.50465 | 1 | 0.40215 | 0.39561 | 1 | 1 | 0.01212 |
| Pyrimidine metabolism | 39 | 0.51793 | 1 | 0.4103 | 0.3869 | 1 | 1 | 0.01318 |
| Primary bile acid biosynthesis | 46 | 0.61089 | 1 | 0.46443 | 0.33308 | 1 | 1 | 0.02239 |
| Aminoacyl-tRNA biosynthesis | 48 | 0.63745 | 1 | 0.47901 | 0.31966 | 1 | 1 | 0 |
| Purine metabolism | 66 | 0.87649 | 1 | 0.59429 | 0.226 | 1 | 1 | 0.01945 |
