# Supplementary material for: Pyrroloquinoline quinone inhibits PCSK9-NLRP3 mediated pyroptosis of Leydig cells in obese mice
Source: Cell Death Dis. 2023 Nov 7;14(11):723. doi: 10.1038/s41419-023-06162-8 (PMC10630350; doi:10.1038/s41419-023-06162-8)
Supplement: Supplementary file 12 — Supplementary Table 5 [file 41419_2023_6162_MOESM12_ESM.docx]

**Table S5. Related parameters of the differential metabolites identified pathways in serum metabolomics analysis (OBEPQQ vs OBE).**

| Index  Pathway | Total | Expected | Hits | Raw p | -log10(p) | Holm adjust | FDR | Impact |
| --- | --- | --- | --- | --- | --- | --- | --- | --- |
| Taurine and hypotaurine metabolism | 8 | 0.079365 | 2 | 0.002486 | 2.6044 | 0.20886 | 0.20886 | 0.42857 |
| Primary bile acid biosynthesis | 46 | 0.45635 | 3 | 0.009282 | 2.0323 | 0.77043 | 0.38986 | 0.04524 |
| Phenylalanine, tyrosine and tryptophan biosynthesis | 4 | 0.039683 | 1 | 0.039134 | 1.4074 | 1 | 0.63087 | 0.5 |
| Glycine, serine and threonine metabolism | 34 | 0.3373 | 2 | 0.042922 | 1.3673 | 1 | 0.63087 | 0 |
| Glycerophospholipid metabolism | 36 | 0.35714 | 2 | 0.047654 | 1.3219 | 1 | 0.63087 | 0.04318 |
| D-Glutamine and D-glutamate metabolism | 5 | 0.049603 | 1 | 0.048692 | 1.3125 | 1 | 0.63087 | 0 |
| Arginine and proline metabolism | 38 | 0.37698 | 2 | 0.052573 | 1.2792 | 1 | 0.63087 | 0.01212 |
| Phenylalanine metabolism | 12 | 0.11905 | 1 | 0.11315 | 0.94634 | 1 | 1 | 0.35714 |
| Biosynthesis of unsaturated fatty acids | 36 | 0.35714 | 1 | 0.30453 | 0.51637 | 1 | 1 | 0 |
| Arachidonic acid metabolism | 37 | 0.36706 | 1 | 0.3116 | 0.5064 | 1 | 1 | 0.32059 |
| Aminoacyl-tRNA biosynthesis | 48 | 0.47619 | 1 | 0.38504 | 0.41449 | 1 | 1 | 0 |
| Steroid hormone biosynthesis | 75 | 0.74405 | 1 | 0.5355 | 0.27124 | 1 | 1 | 0.00786 |
