# Supplementary material for: Pyrroloquinoline quinone inhibits PCSK9-NLRP3 mediated pyroptosis of Leydig cells in obese mice
Source: Cell Death Dis. 2023 Nov 7;14(11):723. doi: 10.1038/s41419-023-06162-8 (PMC10630350; doi:10.1038/s41419-023-06162-8)
Supplement: Supplementary file 13 — Original Data File [file 41419_2023_6162_MOESM13_ESM.docx]

As for images of the original western blots, in order to avoid wasting the gel of which next to the molecular weight of the target protein was cut out for transfer after electrophoresis. Meanwhile, the size of the membrane matches the size of the gel. After the transfer, the membranes are incubated with the corresponding Antibodies, blocked and washed individually, imaged and filmed eventually and the membrane or the images were not cut anymore. Besides, the membrane on the same gel was compared with the same membrane incubated Tubulin. That’s why our membrane is essentially rectangular and some membrane seems to exist with no Tubulin for comparison in the original image.

**Figure 4**

**G** StAR

 Tubulin



P450scc

 Tubulin



3β-HSD

 Tubulin



**Figure 5**

**B** Caspase-1

 Tubulin



GSDMD

 Tubulin



IL-1β

 Tubulin



IL-18

 Tubulin



**Figure 6**

**B** PCSK9



Tubulin





LDLR

 Tubulin



NLRP3



 Tubulin





**Figure 7**

**G** PCSK9



 Tubulin





LDLR


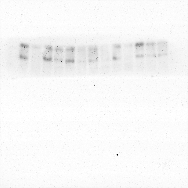
 Tubulin





NLRP3



 Tubulin





StAR



 Tubulin





P450scc



 Tubulin





3β-HSD





Tubulin





Caspase-1



 Tubulin





GSDMD





 Tubulin







IL-1β



 Tubulin





IL-18



 Tubulin
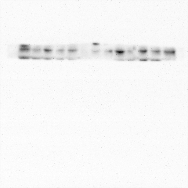




**Figure 8**

**F** NLRP3



 Tubulin





PCSK9



 Tubulin





LDLR





 Tubulin







StAR



 Tubulin





P450scc



 Tubulin





3β-HSD





 Tubulin







Caspase-1





 Tubulin





GSDMD Tubulin

IL-1β Tubulin

IL-18 Tubulin

**Supplementary Figure 4**

**G** PCSK9 Tubulin

LDLR Tubulin

NLRP3 Tubulin

StAR Tubulin

P450scc Tubulin

3β-HSD Tubulin

Caspase-1 Tubulin

GSDMD Tubulin

IL-1β Tubulin

IL-18 Tubulin
